# Effect of biopolymer structure on uranium sorption by superabsorbent hydrogels based on CMC/guar gum

**DOI:** 10.1038/s41598-026-46963-3

**Published:** 2026-04-20

**Authors:** Shimaa M. Elsaeed, Elsayed G. Zaki, Ibrahim E. El-Tantawy, Marwa M. Rashad, Ahmed A. Galhoum

**Affiliations:** 1 Egyptian Petroleum Research Institute, Cairo, 11727 Egypt; 2 Kantra Branch, Sinai University, Center for Scientific Research and Sustainable Development, Ismailia, 41632 Egypt; 3 Faculty of Postgraduate Studies for Nanotechnology, Cairo University, El-Sheikh Zayed, Giza, 12588 Egypt; 4 Chemistry Department, Faculty of Science, Menoufia University, Shebin El-Kom, Egypt; 5 Nuclear Materials Authority, El-Maadi, P.O. Box 530, Cairo, Egypt; 6 Natinal Committee for Women in Science, ASRT, Cairo, Egypt

**Keywords:** Hydrogels, Carboxymethyl cellulose, Guar gum, Graft copolymerization, UO_2_^2+^ sorption, Sorption isotherms and kinetics, Ore leachate application, Chemistry, Environmental sciences, Materials science

## Abstract

Nuclear power provides critical carbon-free energy, while sustainable uranium recycling enhances resource efficiency and reduces waste, requiring advanced selective recovery materials. Herein, superabsorbent hydrogels based on carboxymethyl-cellulose (F-CMC) and guar gum (F-GG) were synthesized via free-radical polymerization. The physicochemical properties were characterized via CHN, SEM, FTIR, TG/DTA, zeta potential, BET surface area analysis, EDX, and swelling properties. Their uranium (UO₂^2^⁺) sorption was evaluated in slightly acidic solutions (optimal pH_0_: 4.0), with F-CMC exhibiting superior capacity (269.26 mg/g, ≈1.131 mmol/g) compared to F-GG (⁓169.38 mg/g, ≈0.712 mmol/g). Sorption isotherms followed Langmuir model, reaching equilibrium in 120 min, with pseudo-first-order kinetics, indicating a combined mechanism of ion-exchange and chelation rather than simple diffusion. The SEM micrographs reveal that F-CMC exhibits surface roughening and granular deposits after sorption, whereas F-GG shows pore filling accompanied by aggregation. Thermodynamic analysis confirmed the process was spontaneous, entropy-driven, and exothermic. Regeneration using 0.25 M NaHCO₃ (or 0.2 M HCl) achieved near-complete uranium desorption and retained ≈80% efficiency over 5 cycles, demonstrating good stability and reusability. Finally, both sorbents effectively recovered U(VI) from acidic El-Sella ore leachates, with F-CMC achieving 0.807 mmol/g and F-GG achieving 0.609 mmol/g, the latter demonstrating superior selectivity in the the complex, multi-ion matrix. The EDX analysis confirmed F-CMC’s superior UO₂^2^⁺ sorption, dominated by K⁺/UO₂^2^⁺ ion-exchange and Al/U co-sorption, whereas F-GG exhibited lower capacity due to competitive Ca^2^⁺ interference.

**Supplementary Information:**

The online version contains supplementary material available at 10.1038/s41598-026-46963-3.

## Introduction

Nuclear power provides carbon-free energy to meet rising global demand. Closed fuel cycles via uranium recycling offer enhanced sustainability through resource efficiency and reduced waste. Recalculating these benefits requires advanced separation materials engineered for selective uranium recovery, a key challenge in nuclear materials science^1–5^. Uranium is essential for nuclear energy programs, but faces scarce natural reserves, with impending shortages soon threatening sustainable operations. Uranium enters the environment via mining, production, or use, threatening health and ecosystems with radioactivity and toxicity. While uranium offers significant benefits, improper use and disposal can lead to its release into the environment, contaminating ecosystems, groundwater, rivers, and oceans. Due to its toxicity, uranium exposure adversely affects wildlife and human health, particularly targeting organs such as the lungs, liver, and kidneys^6,7^. The recommended tolerable daily intake (TDI) for soluble uranium is 0.6 μg/kg of body weight. However, near uranium mining sites, daily intake levels can reach 13–18 μg/d or higher. In contrast, uncontaminated regions typically exhibit uranium intake from food and water at much lower levels, ranging from 1 to 4 μg/d^7^. Concurrently, highly radiotoxic uranium(VI) requires efficient removal/recovery from wastewater to mitigate environmental risks and enable closed fuel cycles. Advanced separation materials are thus critical for maximizing resource utilization while ensuring environmental safety^1,2,8–10^. Efficient separation techniques are essential to address radiological hazards while maximizing resource utilization, ensuring environmental safety alongside energy production. Established uranium extraction techniques include solvent extraction^11^, electrochemical extraction^12^, ion-exchange^8,13,14^, chelation^4^, membrane filtration^15^, photocatalytic uranium extraction^16^, and sorption^1,12,17^. Among these, sorption stands out as the most effective and practical approach due to its operational simplicity, cost-effectiveness, and ease of implementation^8,13,18,19^. The sustainability of nuclear power hinges on safely managing and disposing of radioactive waste produced during spent fuel processing and reprocessing^2,5^. Over the past decade, diverse sorbents for uranium recovery have been engineered, including carbon-based materials^8^, inorganics^5^, polymers^14^, metal − organic frameworks^1^, nanocomposites^9,17,18^, and chelating sorbents^4^. Chelating polymeric systems demonstrate superior effectiveness owing to their high selectivity and affinity for uranium species. This is because their structure customizability, especially in hydrogels, allows targeted functionalization with chelating groups. Grafting these agents onto the polymer backbone significantly enhances sorbent selectivity and sorption capacity for uranium species^14,20–22^.

Natural polymer-based hydrogels derived from plants or marine organisms are promising for U(VI) removal due to their sustainability, cost-effectiveness, and structural tunability, which enable efficient uranyl sorption for eco-friendly resource recovery^21,23–26^. Hydrogel, one of the most widely used polymers, consists of a three-dimensional (3D) hydrophilic polymeric network, leveraging interpenetrating architectures functionalized with chelating groups (e.g., − COOH, − OH, − NH_2_). These engineered structures significantly enhance binding affinity and sorption site density for target species^20,21,27,28^. Carboxymethyl cellulose (CMC), a biocompatible, low-cost, and renewable cellulose derivative, is widely exploited in paper, textiles, food, and pharmaceuticals due to its hydrophilicity and non-toxicity^23,24^. Guar gum (GG), a high-molecular-weight polysaccharide, finds utility in wastewater treatment, cosmetics, pharmaceuticals, and food industries^27,29^, leveraging its structural versatility and sustainability. Both biopolymers serve as sustainable platforms for advanced sorbent design, owing to their oxygen-rich multifunctional groups that enable strong U(VI) binding through diverse interaction mechanisms. Intelligent hydrogels enable efficient heavy metal recovery from wastewater via advantageous properties: high sorption capacity, rapid kinetics, broad pH tolerance, chemical stability, extensive surface area, facile regeneration, stimuli-responsive swelling, and engineered metal-chelation functionality^14,21,27,29^. Many of the current hydrogel sorbents for uranium recovery and environmental remediation often face limitations such as low selectivity, insufficient reusability, and challenges in treating complex acidic leachates from mineral deposits^14,19,22,28,30,31^. While various sorbents have been developed, there remains a significant need for cost-effective, highly selective, and regenerable materials capable of efficiently capturing uranium from complex and competitive matrices.

Based on the above facts, two new superabsorbent hydrogels based on carboxymethyl cellulose (CMC-g-hydrogel) and guar gum (GG-g-hydrogel were synthesized via free radical polymerization reaction to produce F-CMC and F-GG, respectively, for enhanced uranium sorption from aqueous solutions. These materials were characterized via elemental analysis, FTIR, TGA, SEM, zeta potential, BET surface area, swelling studies, and EDX to determine their physicochemical properties and structure. Subsequently, uranium (as uranyl ions ‘UO_2_^2+^’) sorption capabilities were evaluated through pH optimization, kinetic profiling, isotherm modeling, and thermodynamic analysis. Finally, sorbent regenerability and reusability were assessed over multiple cycles, followed by successful validation of uranium recovery using acidic mining effluent.

## Experimentals

### Materials

Chemicals. Carboxymethyl cellulose (CMC), guar gum (GG), acrylic acid (AA), acrylamide (AM), acrylamide-co-2-acrylamido-2-methylpropane sulfonic acid (AMPS), potassium persulfate (KPS), and N,N′-methylenebisacrylamide (MBA) were sourced from Sigma-Aldrich (Germany). Arsenazo III (analytical grade) was obtained from Fluka AG chemicals (Buchs, Switzerland). Other chemicals were Prolabo products used as received. Methods. A 1000 mg/L uranium stock solution was prepared by dissolving uranyl nitrate hexahydrate (UO₂(NO₃)₂·6H₂O) in concentrated H₂SO₄ under heating, followed by dilution with deionized water. Working solutions were freshly diluted before each experiment. Analysis. Uranium concentrations were quantified spectrophotometrically using Arsenazo III indicator^32^ with a Jasco V-570 UV–VIS-NIR spectrophotometer (JASCO, Japan). Deionized water (DI) was used throughout all experiments.

### Preparation of superabsorbent hydrogels

**Scheme 1:**
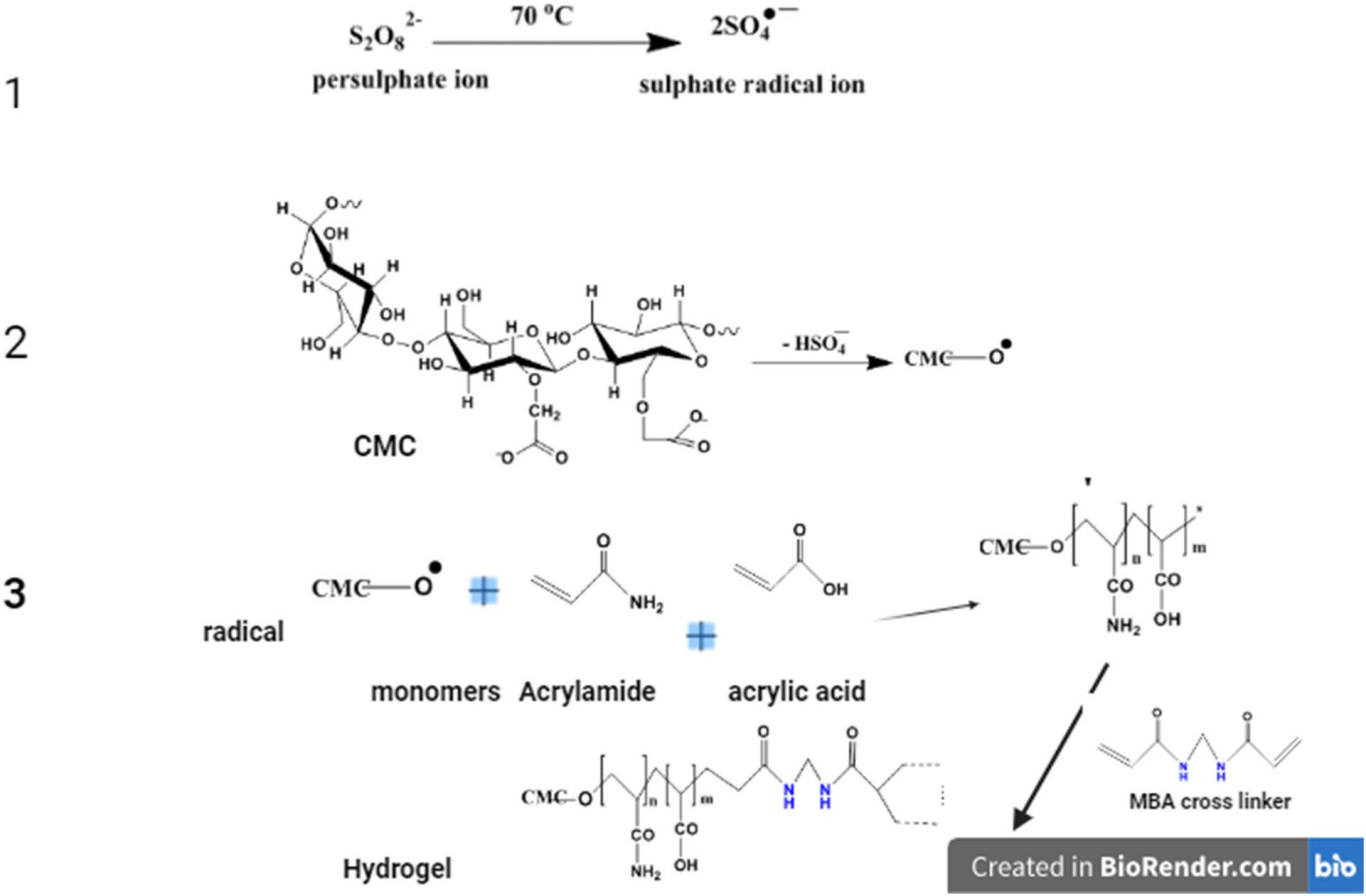
Schematic pathway for superabsorbent hydrogels synthesis.

Scheme 1 provides the visual representation synthesis pathway and the proposed chemical architecture of superabsorbent hydrogels, detailed as follows: Initially, a CMC-g-hydrogel and GG-g-hydrogel were synthesized via free radical polymerization: initially, 1 g CMC or GG was dissolved in 15 mL DI water (50 mL volumetric flask). Subsequently, an aqueous solution of acrylic acid (AA) (12.5 mL, 0.5 g/L) was neutralized by the gradual addition of 4.0 M NaOH to achieve a pH between 10.0 − 10.5, followed by adding acrylamide (AM, 1.0 g). This mixture was then added to the biopolymer CMC/GG solution and homogenized at 40 °C for 30 min to ensure uniform blending. Upon heating to 70 °C, the polymerization initiator, potassium persulfate (KPS, 0.8 g), was introduced, and the mixture was stirred for 15 min under a N₂ atmosphere, after which N,N’-methylenebisacrylamide (MBA, 0.06 g) was added. To ensure complete polymerization, the reaction was polymerized at 70 °C for 3 h with continuous stirring^33^. The optimized composition used biopolymer (5 g), AA (10 mL), AM (2 g), KPS (0.7 g), and MBA (0.06 g). Resulting GG-g-poly(Am-AA) and CMC-g-poly(Am-AA) hydrogels, codded as F-GG and F-CMC, respectively, were fragmented and thoroughly rinsed using sequential washes with hot DI water and ethanol to eliminate any residual unreacted substances. Finally, after overnight drying at 60 °C, materials were ground to 125–250 μm particles using a hammer mill and stored in a desiccator. In intitation step, KPS thermally decomposes to sulfate radical anions (SO₄•–). This anion abstracts -H from C on CMC, generating a CMC-macro-radical. Then add vinyl monomer (AA/Am) to the growing graft chain (CMC-g-poly(AA-co-Am)-Propagation step, then MBA crosslinker reacts with the growing chains to form a 3D-network. The hydrogel yield, calculated using Eq. (1), was 96.1%:

1 $$Yield (\%)=\frac{{(W}_{g})}{{(W}_{0}-M)}$$

where Wg is the final dry hydrogel mass after purification, W_0_ represents the initial dry CMC mass, and M denotes the total mass of monomer and crosslinker, excluding the initiator, water, and base.

### Characterization

The elemental analysis (CHN) was performed at the Micro Analytical Unit of Al-Azhar University (Egypt) using a CHNS Vario EL III automated analyzer (Elementar, Germany). All other characterizations were conducted at the Egyptian Petroleum Research Institute (EPRI). Fourier Transform Infrared (FTIR) spectra were acquired on a Thermo Fisher Nicolet iS10 spectrometer (Waltham, MA, USA) within 4000–400 cm⁻^1^. Surface area and porosity were determined by N₂ adsorption–desorption at 77 K (Quantachrome Nova 3200, Quantachrome Instruments, Boynton Beach, FL, USA), using Brunauer–Emmett–Teller (BET) and Barrett-Joyner-Halenda (BJH) methods, respectively, after degassing samples at 80 °C for 3 h (Belsorp-Max II). TGA was performed on an SDT-Q 600 V20.5 Build 15 apparatus (TA Instruments, Eschborn, Germany) under N₂ atmosphere (20 mL/min) at 25 to 600 °C range, with a heating rate (10 °C/min). Zeta-potential measurements were carried out using a Zetasizer Nano ZS (Malvern Instruments Ltd., UK). Scanning electron microscopy (SEM) observations were carried out using a Prisma E-SEM instrument (Thermo Fisher Scientific Inc., Waltham, MA, USA).

The swelling behavior was evaluated across an initial water pH 2.0–12.0 range, by immersing 0.5 g of dried F-CMC/F-GG hydrogel in 200 mL DI water. Initial pH was adjusted to target values using 0.1/0.5 M/concentrated HCl/NaOH and monitored via pH meter (HANNA pH213, HANNA, USA). After 24 h static immersion at 25 °C to reach equilibrium (Q_m_)^33^, swollen hydrogels were filtered (100-mesh sieve), drained for 20 min, and weighed. The equilibrium swelling ratio was calculated using Eq. (2):

2 $${Q}_{eq}(g/g)=\frac{{(W}_{2}-{W}_{1})}{{W}_{1}}$$

where W_1_ and W_2_ represent dry and swollen weights, respectively.

### Sorption and desorption experiments

Batch tests combined 5 mg of F-CMC/F-GG sorbent with 10 mL of U(VI) solution, with an initial uranium concentration (C_0_: 50 mg/L) in 30 mL glass containers (AS One glassware, Laboratory Bottles Borosilicate with a fit cap). The mixtures were agitated at a rate of 200 rpm at ambient temperature (27 ± 1 °C) for 4 h. Post-equilibrium, solid and liquid were separated, and the residual U(VI) concentration (C_eq_, in mg/L) was quantified via the Arsenazo III method. The equilibrium sorption capacity (q_eq_, mg/g) was calculated using Eq. (3):

3 $${q}_{eq}=\frac{{(C}_{o}-{C}_{eq}) \times V}{m}$$

where V: solution volume (L) and m: sorbent mass (g). All sorption experiments used consistent conditions (detailed in figure captions): equilibrium time = 180 min, temperature = 27 ± 1 °C, pH_0_ = 4.0, and n = 2–3 replicates (standard deviation < 7.8%). Parameters were sequentially investigated: (1) pH effect (1.0–5.0), (2) uptake kinetics (0–180 min), (3) isotherms (27–50 °C), desorption/reusability, and (4) application to an acidic ore leachate. The initial pH (pH_0_) was adjusted using 0.5/0.1 M NaOH or HCl solutions. The equilibrium and kinetic sorption data were analyzed using linearized equations of the models (Table S1, Supplementary Information).

For recycling studies, 20 mg F-CMC/F-GG sorbent was exposed to 50 mL UO_2_^2+^ solution (150 mg/L, 120 min, 26 °C) over five consecutive adsorption–desorption cycles. Post-sorption, desorption was employed using 25 mL 0.05 M NaHCO₃ or 0.2 M HCl (60 min, 25 °C) with stirring, then regeneration via DI water rinse. After each sorption/desorption cycle, UO_2_^2+^ concentrations were spectrophotometrically analyzed. Mass balance closure (± 2%) was confirmed by comparing the initial UO_2_^2+^ concentration with the sum of desorbed uranium in solution and residual uranium on the sorbent surface. Both sorption efficiency (SE) and desorption efficiency (DE) were calculated using the following Eqs. (4 & 5):

4 $$SE=\frac{{(C}_{des} \times {V}_{des})}{{(q}_{ads}\times m)}\times 100$$

5 $$DE=\frac{{(C}_{des} \times {V}_{des})}{{(C}_{des}\times m)}\times 100$$

where C_des_ = eluate concentration (mg/L), V_des_ = eluent volume (L), q_ads_ = sorbed uranium (mg/g), and m = sorbent mass (g). Performance retention was assessed by comparing SE/DE percentages after five cycles to the initial value.

### Sorption testing on acidic uranium ore leachate

The El-Sella mining area is located in the southeastern desert region of Egypt, specifically between latitudes 22°14′30" and 22°18′36" N, and longitudes 36°11′45" and 36°16′30" E, at ⁓60 km southwest of the Abu Ramad city^34^. The ore was acidic leached as follows: sulfuric acid concentration (50 g/L H₂SO₄), particle size (less than 200 mesh), solid:liquid ratio (1:3 S:L), at a temperature (45 ± 2 °C) for a leaching time of 6 h^34^. The resulting leachate had a uranium concentration of ⁓299.32 mg/L, representing a leaching efficiency of 77.69% (Table S2). To evaluate the practical applicability of the sorbents, F-CMC and F-GG were tested for uranium recovery from a complex multi-ion matrix. Before sorption, the acidic leachate pH was adjusted from approximately initially pH: 2.17 underwent partial neutralization to 3.94 via controlled NaOH addition, inducing partial iron precipitation, and reducing the final uranium concentration to 270.19 mg/L. The sorption trials employed 20 mg sorbent per 40 mL pretreated leachate (2 h, 2 6 °C, 200 rpm). Post-equilibrium, the concentrations of the remaining metal ions in the solution were measured. Notably, the uranium concentration was specifically determined using titration^35^.

## Results and discussion

### Characterization section

#### Fourier transform infrared (FTIR) analysis

**Fig. 1:**
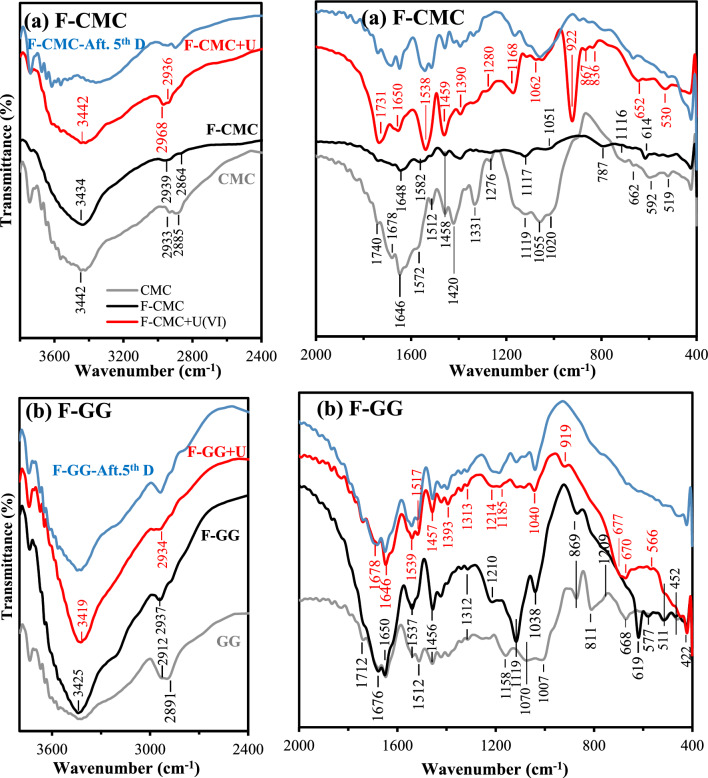
FTIR spectra of CMC and F-CMC: (**a**) pre-/post-sorption, after 5 × desorption, and GG and F-GG: (**b**) pre-/post-sorption, after 5 × desorption.

FTIR spectroscopy characterized F-CMC/F-GG functional groups, confirming synthesis and UO_2_^2+^ interaction mechanism. Fig. 1 presents the FTIR spectra of raw GG/CMC, F-GG/F-CMC pre/post-UO₂^2^⁺ sorption, and after 5 sorption/desorption cycles. Fundamentally, the spectra showed broad similarity, with key differences in peak intensities. The FTIR spectra of raw CMC and GG matrices show a broad absorption band near 3442/3432 cm⁻^1^ (CMC/GG), assigned to − OH stretching and intermolecular/intramolecular hydrogen bonding^33,36^. The doublet peaks at 2930/2912 cm^−1^ and 2881/2891 cm^−1^ for CMC/GG, assigned to asymmetric and symmetric ‘C–H’ stretching vibrations of methylene (− CH₂) groups, respectively^37,38^. The bands at 1647 cm⁻^1^ (CMC) and 1650 cm⁻^1^ (GG) likely arise from adsorbed water (H–O-H bending), with potential contributions from the carboxylate of CMC or residual acetyl groups in raw GG^33,36^. The 1320/1391 cm⁻^1^ and 1605/1650 cm⁻^1^ bands for CMC/GG correspond to primary and secondary –OH stretching vibrations, respectively, which are overlapped with the vibrations of the water molecules^10,39,40^. The weak bands at 1740 cm⁻^1^ band in GG align with acetyl (–COCH₃) vibrations in galactomannan, while the 1738 cm⁻^1^ peak in CMC suggests trace ester impurities or incomplete carboxymethylation. This interpretation is further supported by the 1512 cm⁻^1^ band attributed to protonated carboxyl (–COOH stretching)^33,36,39^. Bands at 1431/1457 cm^−1^ for CMC/GG, (− CH₂ − scissoring), 1331/1390 cm^−1^ for CMC/GG (C − H bending vibrations, overlaps with O–H bending^39,40^), minor peak at 1267 (C–O–C asymmetric stretching assigned to ether linkage in CMC^24^, and/or C − H deformation vibration^41^, 1114/1158 cm^−1^ for CMC/GG, (C–O–C glycosidic ether (β-(1 → 4)-linkage)), 1055/1070 (C-O stretching (secondary alcohols –OH of CMC/GG backbone^42,43^. Weak absorption bands at 1022/1070 cm^−1^ for CMC/GG (C-O stretching (primary -OH of glucose)), 697/668 cm^−1^ for CMC/GG (Out-of-plane -OH bending), 589/576 and 519/512 cm^−1^ for CMC/GG (Skeletal vibrations (C–C-O bending, ring deformation)^38,39^. In GG spectrum, bands at 810 and 869 cm^−1^ correspond to α-(1,6) and β-(1,4)-glycosidic bonds of galactose and mannose, respectively^40^. In F-CMC/F-GG, the spectrum peaks at 3442/3432 cm^−1^, 1540/1538 cm^−1^, and 1459/1456 cm^−1^, indicating the existence of amino functional group^13,18,42^. Successful grafting was confirmed by reduced –OH signal intensity/broadness at 3442/3432 cm^−1^ (for F-CMC/F-GG) versus native CMC and GG spectra, accredited to the − NH_2_ stretching vibration^40,44^. The absorption bands at 1732/1713 cm^−1^ and 1650/1640 cm^−1^ for F-CMC/F-GG (Amide I, C = O stretching) attributed to carboxamide, 1540/1538 cm^−1^ (Amide II, and N–H bending), 1459/1456 cm^−1^ (CH₂ bending or COO⁻ vibrations), and 1390/1365 cm⁻^1^ (Amide III, and C–N of amide) confirm amide (–CONH_2_) functionalization^14,33,42^. The 1275/1209 cm⁻^1^ peak for F-CMC/F-GG corresponds to C–N stretching of amines^33^.

After UO₂^2^⁺ sorption, new bands corresponding to uranyl asymmetric stretching (ν₃) appeared at 867 cm⁻^1^ (F-CMC) and 887 cm⁻^1^ (F-GG)^13^. Additionally, F-CMC spectrum exhibited a characteristic uranyl peak at 836 cm⁻^1^ (symmetric O = U = O stretch) and a sharp band at 923 cm⁻^1^, confirming high sorption efficiency^45,46^. The appearance of a 530 cm⁻^1^ band (U–O-C) further demonstrates inner-sphere complexation. A comparative spectral analysis after 5 cycles revealed minimal changes, demonstrating the sorbent’s exceptional stability and consistent sorption/desorption performance. FTIR analysis (Fig. 1) showed partial spectral recovery after desorption, with residual metal-related bands persisting in the regenerated sorbents. These ‘intermediary’ spectra suggest incomplete elution, exhibiting characteristics between raw and metal-loaded states^37^.

#### CHN analysis

Elemental analysis in weight percent (wt%, Table S3) confirms successful functionalization of CMC/GG biopolymers via graft copolymerization, as evidenced by substantial nitrogen enrichment in F-CMC (10.48% *vs.* 0.12% in raw CMC) and F-GG (12.46% *vs.* 0.88% in raw GG). This 87-fold N-increase in CMC and 14-fold in GG directly corresponds to the incorporation of nitrogen-rich acrylamide/AMPS monomers. Carbon trends diverge: F-CMC shows a slight C-decrease (36.56% *vs.* 37.27%), while F-GG exhibits a marginal C-increase (40.21% *vs.* 39.89%), suggesting differential grafting densities. Hydrogen content decreased in both functionalized sorbents (F-CMC: 5.56% *vs.* 5.98%; F-GG: 6.79% *vs.* 6.95%), consistent with hydrocarbon backbone modifications, taking into account the theoretical initial and final molar mass.

#### Thermal properties-TG/DTA analysis

**Fig. 2:**
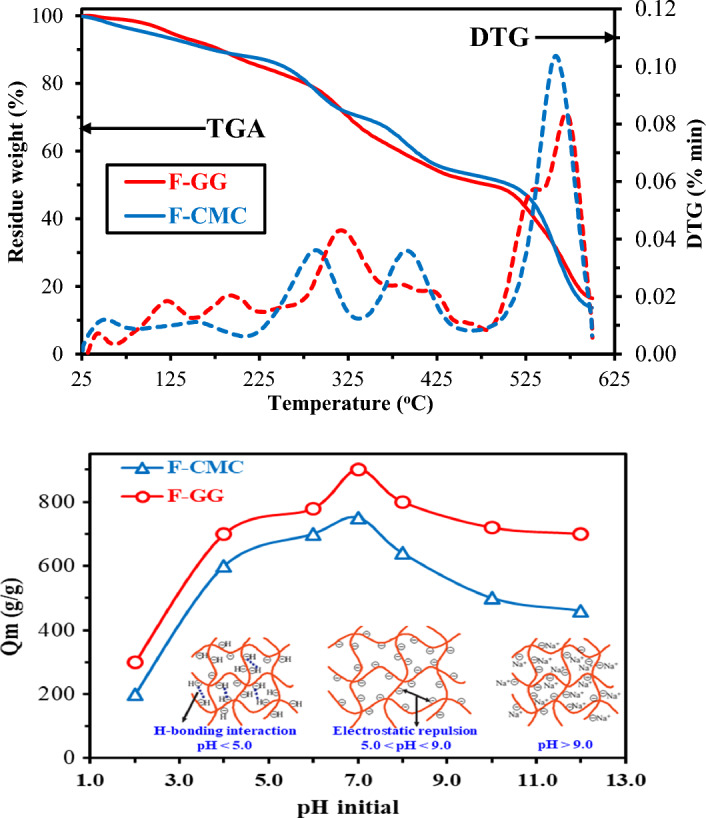
Thermogravimetric analysis: TGA curves (Solid line) and DTG curves (dotted line): (Wt. loss% = 100%—wt. residue%) (Upper panel) and Water absorption and structure models of superabsorbent hydrogel at different pH levels (Lower panel).

Figure 2(Upper pannel) presents the results of thermogravimetric analysis (TGA) profiles of F-CMC and F-GG sorbents under N_2(g)_ environment. Both materials exhibit highly similar degradation profiles, featuring five overlapping mass loss stages across comparable temperatures with progressively slower mass loss. The five degradation transition stages^19^ are as follows: a) the initial stage (25–198 °C) corresponds to the evaporation of surface-adsorbed water and a significant portion of structurally bound, particularly interlayer water, resulting in ~ 12% mass loss. Subsequently, b) stage two (198–329 °C) results in an additional decomposition of amine functionalities (inherent to active chelating moieties components) derivatives within the functionalized sorbents, contributing an additional ~ 17% loss (giving cumulative weight loss to ~ 29%), c) stage three (329–437 °C) shows a decelerated mass loss rate (~ 15–18%), indicative of altered pyrolysis mechanisms, primarily attributed to pyranose ring cleavage; this yields a cumulative mass reduction of ~ 45–46%, and d) the fourth stage (437–525 °C) exhibits further decomposition and additional weight loss (~ 8–10% loss), culminating in ~ 53–55% total mass loss by 525 °C, where primary pyrolysis reactions conclude^19,41^. Finally, e) stage five (525–600 °C) features rapid mass loss due to carbonaceous char decomposition. Residual masses at 600 °C were 86.3% for F-CMC and 83.5% for F-GG, confirming their enhanced thermal stability. The congruent TGA profiles further indicate no significant differences in the core chemical structures of the hydrogels^36,41^.

Moreover, the DTG curves (Fig. 2(upper panel) reveal distinct degradation patterns: F-CMC displays five decomposition events, with weak valleys (51–158°C), strong valleys (293–397°C), and maximal degradation at 562 °C (0.09–0.12% min⁻^1^ mass loss rate), corresponding to CMC backbone breakdown. F-GG exhibits seven valleys, including weak (33–199°C), strong (318°C), and intense dual peaks at 536–569°C, but with lower mass loss rates (0.06–0.09% min⁻^1^) reflecting slower galactomannan scission. The sharp, high-temperature degradation of F-CMC contrasts with F-GG’s broader, multi-stage profile, underscoring their structural differences—CMC’s abrupt decarboxylation versus guar gum’s gradual chain depolymerization. These kinetics correlate with the polymers’ functional group stability and backbone robustness under thermal stress.

#### Zeta-potential measurements

Fig. S1 presents the zeta potential analysis for F-CMC and F-GG sorbents at pH 4.0. Both materials exhibit negative surface charges, but with distinct profiles. F-CMC displays a bimodal pattern, with a dominant peak (~ 59.6%) at –34.9 mV and a secondary peak (~ 40.4%) at –18.2 mV, yielding an average of –28.5 ± 11.1 mV at 0.521 mS/cm conductivity. F-GG exhibits a trimodal distribution, with peaks at –37.5 mV (45.2%), –57.5 mV (34.4%), and + 7.91 mV (12.1%), averaging –36.6 ± 22.4 mV at 0.102 mS/cm. The greater negative charge density of F-CMC (−28.5 mV *vs.* −36.6 mV for F-GG) arises from abundant surface carboxylate groups (-COO⁻) introduced via carboxymethyl cellulose functionalization^24^. In contrast, F-GG’s charge heterogeneity, including positive domains, reflects its non-ionic polysaccharide backbone and lower functional group density^27,29^, consistent with elemental analysis showing reduced nitrogen content (12.46% vs. 10.48% for F-CMC).

#### Swelling equilibrium as a pH-dependent

**Fig. 3:**
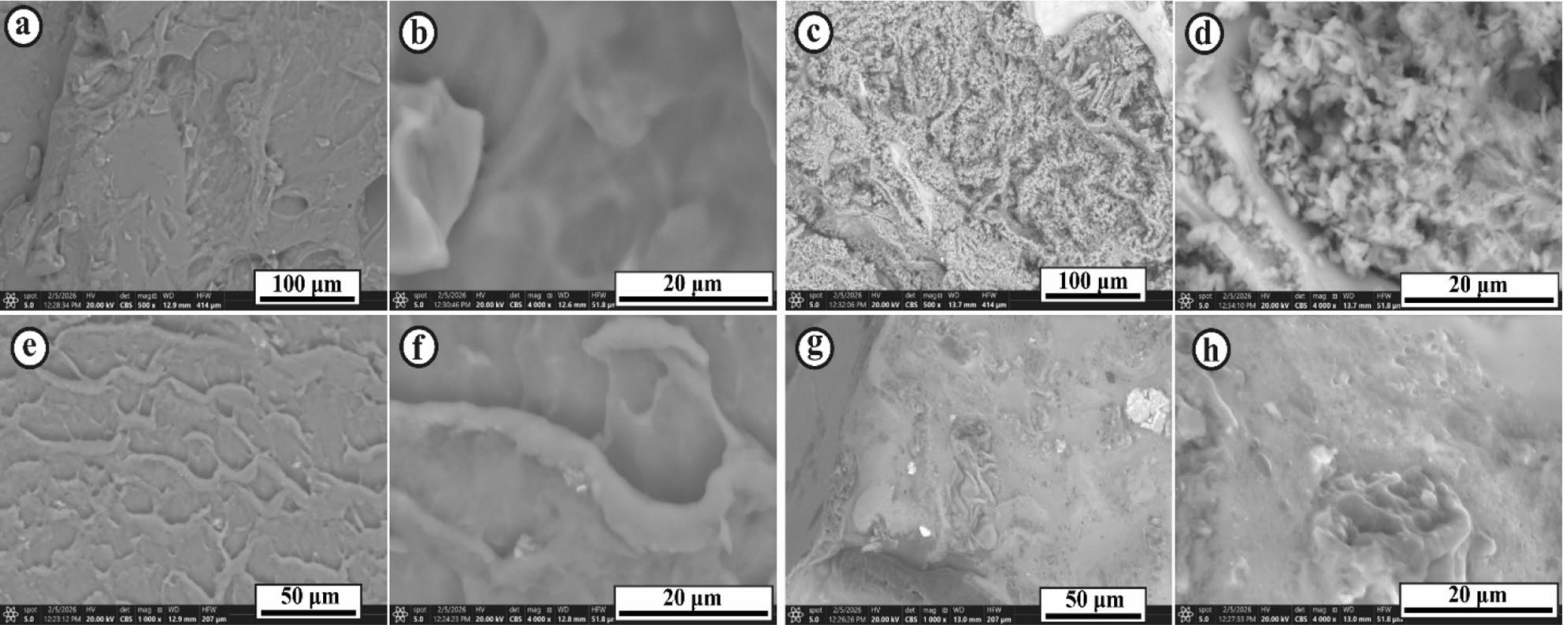
SEM micrographs of (**a, b**) F-CMC before, (**c, d**) F-CMC after sorption, (**e, f**) F-GG before, and (**g, h**) F-GG after sorption at different magnifications.

Anionic superabsorbent hydrogels, F-CMC and G-GG sorbents, commonly feature by carboxylate, carboxamide, and sulfonate groups. These groups confer pronounced pH sensitivity on the swelling behavior of hydrogel sorbents. Equilibrium swelling capacity was therefore measured across a pH range (Fig. 3(Lower panel)). To isolate pH effects and avoid ionic strength interference from buffers, pH was adjusted using distilled water dilutions of HCl (pH 2.0) or NaOH (pH 12.0)^16^. Equlibrium sorption capacities reached 700 g H₂O/g for F-CMC and 900 g H₂O/g for F-GG, at pH 7.0 and 26 ± 1°C^33^. The swelling is governed by the ionization state of anionic groups and their consequences: a) at acidic pH (pH < 5.0): the majority of carboxylate and sulfonate groups are predominantly protonated (–COOH, –SO₃H). This promotes hydrogen bonding between groups, creating additional physical crosslinks within the network. Simultaneously, reduced electrostatic repulsion (–COO⁻, –SO₃⁻) also allows network contraction, significantly decreasing swelling. b) at neutral/mildly alkaline pH (pH 5.0–9.0): progressive ionization of carboxylate and sulfonate groups occurs. This ionization disrupts hydrogen bonds and increases electrostatic repulsion between the resulting anions (–COO⁻, –SO₃⁻). The combined effect causes polymer chain expansion and equilibrium hydrogel swelling, and finally, c) at a highly alkaline pH (pH over 9.0): Swelling capacity decreases, due to the effect of charge screening resulting from a surplus of sodium ions within the solution. The high concentration of Na⁺ ions in solution screens the negative charges on the polymer chains (–COO⁻, –SO₃⁻). This charge screening reduces the electrostatic repulsion driving network expansion, thereby limiting swellability^33,47^.

#### Surface area analysis

The BET analysis reveals critical textural differences between sorbents. Fig. S2 displays N₂_(g)_ adsorption/desorption isotherms, revealing that both exhibit comparable a BET multipoint specific surface areas (S_BET_: F-CMC: 52.16 m^2^/g and F-GG: 51.12 m^2^/g), F-CMC possesses significantly higher pore volume (0.044 cm^3^/g *vs.* F-GG’s 0.015 cm^3^/g). This structural divergence stems from their polymer backbones: the bulky phenolic groups in F-GG create steric hindrance leading to denser packing, whereas F-CMC’s methoxy groups allow a more open network^48,49^. Despite having comparable surface areas, the sorbents differ in their pore architectures. F-CMC features micropores (1.67 nm, < 2 nm diameter rang^50^), which sterically confine hydrated UO₂^2^⁺ species diameter (5.3 Å, equivalent to 0.53 nm)^51^, enhancing selective uptake. Conversely, F-GG’s larger mesopores (6.06 nm, within 2–50 nm range^50^) facilitate faster diffusion but offer less selectivity. This critical pore-size disparity, combined with F-CMC’s tighter crosslinked network (indicated by lower swelling), explains its significantly higher U(VI) capacity (269 mg/g vs. 169 mg/g for F-GG). The distinct nanoscale structures ultimately govern uranyl ion accessibility.

#### Morphological analysis-SEM analysis

SEM micrographs reveal distinct morphological changes in both functionalized hydrogels before and after U(VI) sorption. Pristine F-CMC (Fig. 3a&b) displays a compact, smooth, and relatively continuous surface with limited porosity, showing only minor wrinkles and shallow depressions, indicative of a dense crosslinked matrix. This morphology suggests that sorption sites are primarily internal, accessible mainly through hydrogel swelling rather than surface porosity. Following U(VI) sorption (Fig. 3c,d), F-CMC develops a rough, heterogeneous surface with widespread granular coverage and dense nodular agglomerates, providing direct evidence of uranyl binding and surface accumulation. In contrast, pristine F-GG (Fig. 3e&f) exhibits a highly wrinkled, sponge-like architecture with interconnected cavities and channels, forming a loosely packed, open polymeric network that promotes rapid ion diffusion and ensures functional groups are accessible throughout the matrix. After U(VI) sorption (Fig. 3g,h), F-GG’s surface becomes denser, with pores partially blocked by bright aggregates, confirming deep intraparticle diffusion and immobilization of U(VI). Overall, SEM analysis shows that F-CMC undergoes surface-dominated deposition, whereas F-GG experiences both pore filling and surface aggregation. The more pronounced morphological transformation in F-CMC compared to F-GG underscores its higher sorption efficiency and effective uranyl uptake.

### Uranium sorption properties

#### Influence of pH

**Fig. 4:**
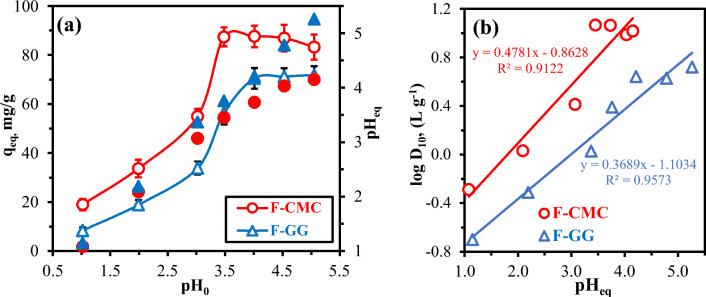
pH effect on UO_2_^2+^ sorption by F-CMC/F-GG: capacity vs. initial pH (closed symbols), equilibrium vs. initial pH (open symbols) (**a**), and log*D*_10_ vs. pH_eq_ (**b**). (C_0_: 50 mg/L, SD: 0.5 g/L, Time: 12 h, temperature (27 °C), 200 rpm).

Solution pH dictates metal sorption affinity by controlling: sorbent surface charge via functional group protonation equilibria, and metal ion speciation and ionic charge. This dual influence regulates Coulombic interactions, complexation, and ion-exchange efficacy^27,29,52^. The UO₂^2^⁺ sorption efficiency was systematically examined across the initial pH (pH_0_) range of 1.0 to 5.0, with corresponding equilibrium pH (pH_eq_) monitoring. At above pH ~ 5.5, UO₂(OH)₂·H₂O colloidal precipitation destabilizes systems, confounding sorption^13,25^. For both sorbents, sorption gradually rises from pH_0_: 1.02 to 3.05, then surges sharply between pH_0_: 3.02–3.48 (Fig. 4). Above pH_0_: 4.01, sorption capacities plateau with a marginal decline, indicating near-saturation of binding sites. F-CMC achieves peak capacity (87.36 mg/g at pH_0_: 3.48) due to carboxyl deprotonation (-COOH to -COO⁻), enabling efficient ion-exchange and correlating with sharp proton release (ΔpH ≤ −0.50 above pH_0_: 3.5) and a plateau (~ 87.31 mg/g) until pH_0_: 4.53. In contrast, F-GG shows a linear increase in sorption (from 8.42 to 71.88 mg/g at pH_0_: 1.02–5.05), driven by progressive proton uptake (ΔpH + 0.13 to + 0.35) via UO_2_^2+^ coordination with hydroxyl groups (-OH). F-CMC outperforms F-GG by > 50% below pH_0_: 3.5, narrowing to ~ 15% at higher pH, highlighting carboxyl groups’ superiority in acidic conditions^10,19,23,25^. Generally, high-capacity uranyl binding across pH_0_: 3.48–5.05 indicates coordinated involvement of multiple functional groups (e.g., amines, amidoxime, carboxyl, and hydroxyl sites), compensating for UO_2_^2+^ speciation shifts and electrostatic repulsion effects. Under acidic conditions (pH_0_ < 4.0), UO_2_^2+^ dominates aqueous speciation, enabling direct interactions with sorbent functional groups^22^. As pH increases to 4.0–5.5, mixed cationic species emerge, including UO_2_^2+^, UO₂(OH)⁺, (UO₂)₃(OH)₅⁺, and (UO₂)₂(OH)₂^2^⁺. Neutral conditions (pH: 5.5–8.0) favor polynuclear (UO₂)₃(OH)₅⁺ and (UO₂)₄(OH)₇⁺ species^10,34^, while sulfate promotes anionic UO₂(SO₄)₂^2^⁻ formation^17,22^. Optimal sorption occurs at pH_0_: 4.0, balancing UO₂^2^⁺ predominance, sufficient functional group deprotonation (-COO⁻), and prevention of colloidal UO_2_(OH)_2_ precipitation (pH_0_ > 5.5).

Moreover, zeta potential analysis (Fig. S1) confirms enhanced electrostatic attraction/complexation above pH_0_: 4.0 as both sorbents develop more negative surfaces^52,53^. F-CMC achieves higher uranium capacity (87.24 vs. 70.50 mg/g for F-GG), attributed to its multifunctional architecture with hydroxyl, carbonyl, carboxyl, and methoxy groups^41,54^. In contrast, F-GG contains only hydroxyl and phenolic moieties^27,29,49^. According to Hard-Soft Acid–Base (HSAB) theory, F-CMC’s hard carboxyl and oxygen donors preferentially coordinate UO₂^2^⁺ (hard Lewis acid), while F-GG’s phenolic -OH groups, softened by mesomeric effects (-M) and steric hindrance, bind less effectively. Additionally, methoxy groups exert electron-donating (+ I) effects in F-CMC enhance electron density, strengthening oxygen donor hardness. This structural advantage accounts for F-CMC’s consistently superior uptake across pH ranges^13,48^.

The pH-dependent trends show contrasting UO₂^2^⁺ sorption mechanisms between F-CMC and F-GG. F-CMC (Fig. 4a) exhibits initial slight pH increases (ΔpH + 0.05 to + 0.06 at pH_0_: 1.02–3.02), suggesting minor proton consumption, followed by sharp decreases (ΔpH −0.03 to −0.90 above pH_0_: ~ 3.5) due to carboxyl group (-COOH) proton release during ion exchange. Conversely, F-GG maintains steady pH rises (ΔpH + 0.13 to + 0.35) across all pH_0_, indicating consistent proton uptake via UO₂^2^⁺ coordination with hydroxyl groups. F-CMC’s transition near pH_0_: ~ 3.5 matches its carboxyl pKa, while F-GG’s stable trend reflects hydrolysis-dominated sorption. The linear pH_eq_ *vs.* logD_10_ relationships (Fig. 4b) confirm pH-controlled sorption, with F-CMC’s steeper slope (0.4781 vs. 0.3689 for F-GG) highlighting stronger pH responsiveness from carboxylate-mediated ion-exchange, while F-GG’s flatter trend aligns with hydroxyl-dominated complexation. It 0.24-unit logD_10_ offset and higher capacity (87.24 vs. 70.50 mg/g) demonstrate its affinity advantage from -COO⁻ chelation^17,53^. Sub-unity slopes indicate non-stoichiometric H⁺ exchange, governed by low-pH proton competition and high-pH U(VI) hydrolysis (e.g., (UO₂)₃(OH)₅⁺ formation). The strong R^2^ values (0.912–0.957) validate pH as the dominant control parameter.

#### Uptake kinetics

**Fig. 5:**
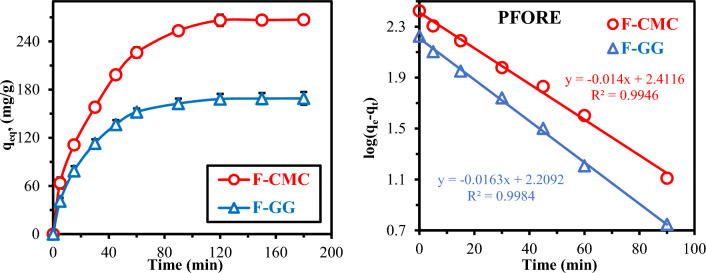
UO₂^2^⁺ kinetics on F-CMC and F-GG ─ PFORE model. (pH_0_: 4.06, C_0_: 200 mg/L, SD: 0.5 g/L, Temperature: 27 °C, 200 rpm).

Figure 5 presents the time-dependent UO₂^2^⁺ uptake, revealing biphasic kinetics, characterized by an initial rapid uptake phase (74.55–81.12% of totalcapacity within first 45 min) driven by abundant surface functional groups (-COOH/-OH in F-CMC; -OH/phenolic in F-GG) and strong concentration gradients, followed by a slower diffusion-limited phase reaching equilibrium within 90–120 min^25,35,47^. Similar initial sorption rates indicate comparable accessibility of surface sites, while the plateau reflects progressive saturation of macropore and mesopore sites through rapid sorption, followed by slower intraparticle diffusion into micropores and internal domains^17,23,25^. F-CMC achieves a 5–7% higher equilibrium capacity, attributed to stronger –COO⁻ chelation compared with hydroxyl-dominated coordination in F-GG. Its higher surface area (52.2 m2/g) and narrower pores (1.67 nm) promote slightly faster uptake, whereas F-GG’s wider pores (6.06 nm) favor gradual diffusion (Fig. 4&S4). The equilibrium at 120 min results from diminishing concentration gradients (ΔC → 0), active site saturation (θ → 1), and established sorption–desorption equilibrium (kₐdₛ[C] = kdₑₛ[UO₂^2^⁺-sorbent])^55–57^. Mechanistically, the kinetics transition from pseudo-first-order surface control to diffusion-limited transport, confirming 120 min as the optimal operational contact time.

The uptake kinetics are governed by three key factors: intraparticle diffusion resistance film diffusion limitations, and reaction rates influenced by hydration dynamics and pH-dependent charge neutralization. The sorption behaviors were modeled using pseudo-first-order (PFORE), pseudo-second-order (PSORE), and simplified resistance to intraparticle diffusion (sRIDE, via Weber-Morris plots) approaches (Table S1)^55–57^. From Fig. 5b & S3 and Table S4, PFORE provided the best fit, exhibiting higher correlation coefficients (R2 > 0.99) compared with PSORE (R2 < 0.99) and more accurate predictions of equilibrium capacity (Δqₑ = 3.11–3.77% deviation versus PSORE overestimation of 8.08–10.45%)^58^. The strong agreement with PFORE indicates that sorption at surface functional groups primarily controls the initial sorption rate, with minimal bulk diffusion resistance during early stages. This behavior suggests an initial physisorption step followed by chemisorption at active binding sites. Finally, the multi-linear *q*_(*t*)_ vs. t^0.5^ plot (Fig. S4) reveals three sequential stages: rapid surface sorption (*K*_*id*,1_​), gradual intraparticle diffusion (K_id,2_​), and final equilibrium (K_id,3_≈0). The higher magnitudes of K_id,1_​ and K_id,2_​ (Table S4) confirm faster initial sorption kinetics, with the subsequent plateau in the third stage indicating the attainment of sorption–desorption equilibrium^37^. The non-zero intercept in the second stage suggests boundary layer effects, confirming that sorption is governed by coupled film diffusion and intraparticle transport mechanisms^55^.

The kinetic analysis indicates a mixed sorption mechanism. While the PFORE model fits the data marginally better, both PFORE (often linked to physisorption) and pseudo-second-order (PSORE, typically for chemisorption) are applicable^55^. This equivalence suggests UO_2_^2+^ sorption is not purely physical or chemical, a conclusion supported by FTIR evidence of post-sorption coordination. The dominant mechanism likely shifts with pH, depending on uranyl speciation and functional group availability.

#### Sorption isotherms and thermodynamics

**Fig. 6:**
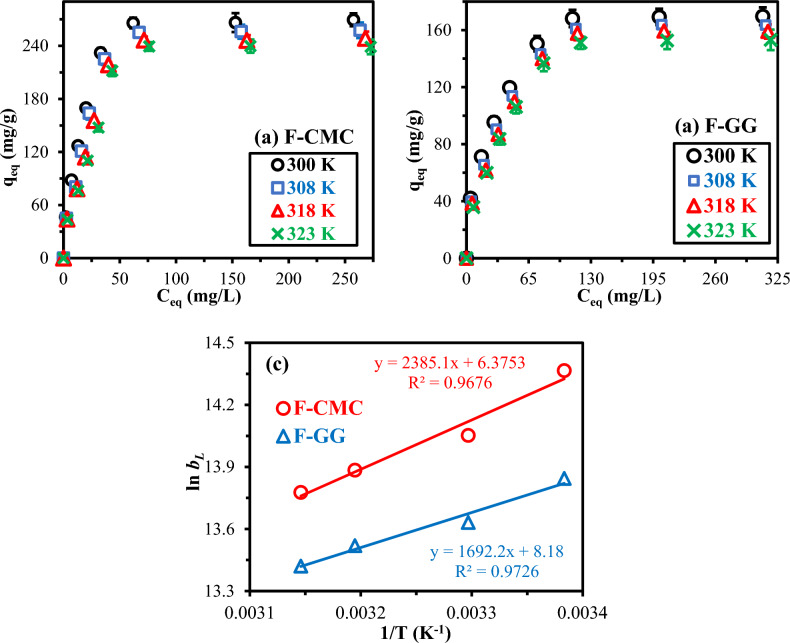
UO_2_^2+^ sorption isotherms on F-CMC (**a**) and F-GG (**b**) at varying temperatures, and (**c**) thermodynamics–van’t Hoff plots (ln *b*_*L*_* vs* 1/T). (pH_0_: 4.06, SD: 0.5 g/L, Time: 2 h, Temperature: 27–50 °C, 200 rpm).

Uranyl sorption isotherms for F-CMC and F-GG were evaluated at four temperatures (27 ± 1°C–55 ± 2 °C) under standardized conditions (pH_0_: 4.1, sorbent dose 0.5 g/L, 200 rpm, 240 min saturation time). The qₑ–Cₑ isotherm profiles (Fig. 6) showed steep initial slopes followed by asymptotic plateaus, indicating rapid surface uptake driven by concentration gradients and subsequent monolayer saturation. F-CMC consistently exhibited faster initial uptake and higher affinity, in agreement with the pH-dependent and time trends discussed earlier. The maximum capacities reached 266.26 mg/g for F-CMC and 169.28 mg/g for F-GG. The superior performance of F-CMC is attributed to a higher density of carboxyl/carbonyl groups enabling strong UO₂^2^⁺ chelation, improved mesoporosity that facilitates intraparticle diffusion, and enhanced accessibility of multifunctional binding sites (–COOH, –OH, –OCH₃). While F-GG benefits from phenolic/hydroxyl moieties that enhance coordination site availability and lower diffusion resistance^14,23,25^. The asymptotic isotherm profiles reveal initial physisorption dominance at low Cₑ (steep slopes) transitioning to chemisorption stabilization near saturation (plateaus), with thermal trends confirming competitive adsorption–desorption equilibria.

The sorption equilibrium data were analyzed using the linear forms of the Langmuir, Freundlich, and Temkin isotherm models (Table S1). While the Langmuir model describes monolayer saturation on homogeneous sites, the Freundlich equation represents heterogeneous, power-law sorption^30,31,59^. Model performance was assessed using R2 and comparison of predicted versus experimental capacities (Table S5; Fig. S5a&b). The Langmuir model provided a markedly superior fit (R2 > 0.99) compared with the Freundlich model (R2 < 0.97), consistent with the asymptotic saturation behavior observed experimentally. For both hydrogels, Langmuir-predicted qₘ values closely matched experimental capacities (e.g., 277.78 vs. 266.26 mg/g for F-CMC at 300 K, < 13% deviation), whereas the Freundlich model significantly underestimated capacities (83–89%). The decline in Langmuir constants with increasing temperature (e.g., b_L_ decreasing from 93.51 to 50.0 × 10⁻3 L/mg for F-CMC from 300–328 K) confirms exothermic monolayer sorption with reduced affinity at higher temperatures. Additionally, separation factors (R_L_ = 1/(1 + b*C*_0_)) ranged from 0.05–0.09 for F-CMC and 0.03–0.05 for F-GG, indicating highly favorable UO₂2⁺ sorption under the studied conditions.

The Temkin isotherm describes sorption as a function of surface-coverage–dependent sorption energy. (Fig. S5c)^60^. For both sorbents, the initial heat of sorption (A_T_) decreases with temperature (F-CMC: 1.5459–0.6230 L/mg; F-GG: 0.9513–0.6578 L/mg), confirming exothermic behavior^44^. Higher A_T_ values for F-CMC indicate stronger initial sorbate affinity than F-GG, aligning with Langmuir trends. The sorption heat (b_T_) shows consistent increases for F-GG (31.96–34.35 kJ/mol), while F-CMC exhibits irregular fluctuations (50.61–52.90 kJ/mol), likely due to the model’s poorer fit (R^2^: 0.848–0.895 vs. Langmuir’s > 0.987). Though Temkin suggests chemisorption contributions (b_T_ > 20–40 kJ/mol), Langmuir’s superior R^2^ values prioritize monolayer sorption as the dominant mechanism.

The full sorption isotherms were systematically evaluated at specific temperatures (27 °C and 50 °C) to more precisely assess the thermodynamic constants (Fig. 6c). The temperature-dependent qₘₐₓ values decreased exothermically for both sorbents: F-CMC declined from 266.26 mg/g at 27 °C to 238.84 mg/g at 50 °C, while F-GG decreased from 169.28 mg/g to 152.68 mg/g, respectively, demonstrating consistent 4–10% capacity reduction at elevated temperatures reflects modest exothermic behavior. The experimental data were fitted using the Langmuir model, with affinity coefficients (b_L_, mg/g, in Table S5. Thermodynamic parameters were derived by converting b_L_ to molar units and applying the Van’t Hoff equation (Eq. 6a). This yielded enthalpy (ΔH°, kJ/mol) and entropy (ΔS°, J/mol.K) changes, from which Gibbs free energy (Eq. 6b) was calculated, providing complete thermodynamic characterization^44,61,62^.

6a $$\Delta G^\circ = \Delta H^\circ -T \Delta H^\circ$$

6b $$\mathrm{ln}{K}_{eq}^{0}= -\frac{\Delta H^\circ }{R} \times \frac{1}{T} + \frac{\Delta S^\circ }{R}$$

Figure 6c shows linear van’t Hoff plots (ln b_L_ *vs.* 1/T), confirming a strong data fit and enabling calculation of ΔH°, ΔS°, and ΔG° (Table 1). The negative ΔH° values (−19.83 kJ/mol for F-CMC; −14.07 kJ/mol for F-GG) confirm exothermic sorption^63^. The more negative enthalpy for F-CMC suggests stronger binding interactions compared to F-GG^63^, consistent with its superior sorption capacity. Positive ΔS° values (16.28 J/mol·K for F-CMC; 31.28 J/mol·K for F-GG) suggest increased randomness, likely from structural/dehydration reorganization^44,64^. The negative ΔG° values (ranging from −24.71 to −25.09 kJ/mol for F-CMC and −23.45 to −24.17 kJ/mol for F-GG) demonstrate spontaneous sorption, becoming more favorable with increasing temperature. The excellent R^2^ values (> 0.96) validate the thermodynamic consistency. The TΔS° terms (4.88–5.26 kJ/mol for F-CMC; 9.39–10.17 kJ/mol for F-GG) reveal entropy-driven contributions are more significant for F-GG, while F-CMC’s process is more enthalpy-dominated.

**Table 1:** Thermodynamic parameters of UO_2_^2+^ sorption.

Sorbent	F-CMC (R^2^: 0.9676)				F-GG (R^2^: 0.9726)			
Temp.w (K)	∆H^o^ (kJ/mol)	∆S^o^ (J/mol)	∆G^o^ (kJ/mol)	T∆S^o^ (KJ/mol)	∆H^o^ (kJ/mol)	∆S^o^ (J/mol)	∆G^o^ (kJ/mol)	T∆S^o^ (KJ/mol)
300	−19.83	16.28	−24.71	4.88	−14.07	31.28	−23.45	9.39
308			−24.84	5.01			−23.7	9.64
318			−25.01	5.18			−24.02	9.95
323			−25.09	5.26			−24.17	10.17

#### Comparison of UO_2_^2+^ sorption by various sorbents

Table 2 provides a comparative assessment of UO₂^2^⁺ sorption capacities across diverse materials, though methodological variations (pH, concentration, dosages, aqueous composition, temperature) preclude direct quantitative comparisons. Table 2 reveals F-CMC as a top-tier sorbent, achieving > 260 mg/g capacity with rapid kinetics, attributable to its multifunctional matrix (–COO⁻/–OH/–OCH₃ groups) that enables efficient U(VI) chelation. While F-GG performs comparably to conventional sorbents (e.g., activated carbons), specialized materials like GA-GT/PAAm resin (348 mg/g at pH 5)^19^ and EPS (333 mg/g at pH 6)^65^ surpass F-CMC under optimized conditions, yet lack its balanced performance across broader operational ranges. Notably, F-CMC’s combination of high capacity and fast kinetics makes it particularly suitable for practical uranium recovery applications where both metrics are critical. This analysis highlights the need for standardized testing protocols to enable rigorous cross-material evaluations, while demonstrating that F-CMC remains competitive even when compared to high-capacity niche sorbents. The results underscore how functional group diversity and accessibility govern sorbent efficacy, with F-CMC’s structural advantages translating to robust real-world performance.

**Table 2:** Comparison of sorption capacity for U(VI) ions with various sorbents.

Sorbent	pH_0,_ T_eq_ (min), Temp. (K)	Q_max._, mg/g	Thermodynamics	Ref
Α-Aps: R-COOH, R-NH_2_, R-H	4.0, 120/180, 298	126.0/2950	Exo-	^4^
AO-NIAMP	6.0, 90, 303	36.30		^28^
GA-GT/PAAm GT/PAAm hydrogel	5.0, 150, 328	348.6 288.9	Endo-	^19^
P(NIPAAm/MA) hydrogels	4.0, 420, 300	94.8	––	^22^
CS-tripolyphosphate beads	5.0, 80 h, 293	236.9	Exo-	^43^
Cyst.MCNPs	3.6 −5.0, 60, 298	97.9	Exo-	^9^
EEPS	6.0, 120 h, 298	333.3		^65^
PPA-PGMA/Fe_3_O_4_	3.5–5.0, 90, 298	266.1	Exo-	^34^
F-GG F-CMC	4.0, 120, 300	169.27 269.26	Exo-	*Here*

*Thermodynamic nature: Exo-: Exthothermic and Endo-: Endothermic,.

#### Uranyl interaction mechanism

##### FTIR characterization

FTIR spectroscopy was employed to identify the functional groups involved in UO₂^2^⁺ binding (Fig. 1). Both F-CMC and F-GG exhibited band shifts, intensity changes, and the emergence of new peaks after sorption, indicating strong chemical interactions and structural rearrangement^4,37^. In F-CMC, shifts in –OH/–NH, carbonyl, and amide bands, together with uranyl-specific vibrations and low-frequency U–O/N bands, confirm coordination with oxygen- and nitrogen-donor groups and inner-sphere complex formation. For F-GG, similar shifts in hydroxyl, carbonyl, and amide regions, along with new U–O/N and uranyl stretching bands, were observed. The disappearance of several native polymer peaks in both sorbents further suggests structural reorganization upon uranium uptake. The FTIR results demonstrate that UO₂^2^⁺ sorption occurs through combined chelation, electrostatic interactions, and hydrogen bonding involving –OH, –NH, and carbonyl/carboxylate groups^8^. Further spectral interpretation is provided in Section B.5 of the Supplementary Information.

##### The elemental composite analysis-EDX characterization

EDX analysis of the F-CMC and F-GG sorbents before and after acidic ore leachate treatment confirmed substantial uranium sorption (Fig. S6 and Table S6). Characteristic uranium signals (at 3.17 keV (Mα₁), 13.44 keV (Lα₂), and 13.62 keV (Lα₁)^4,8^) verified successful sorption, with F-CMC achieving significantly higher loading (21.46 wt%) than F-GG (6.05 wt%), by 3.5 × times, consistent with batch sorption results. The presence of sulfur and iron signals indicates participation of uranyl–sulfate species and competitive sorption of ore-derived ions, while the disappearance of potassium after treatment confirms its role in ion-exchange processes. These observations support a dual sorption mechanism involving direct coordination of UO₂^2^⁺ to protonated functional groups and anion exchange with sulfate complexes under acidic conditions^18,66^. The coexistence of uranium and sulfate signals implies potential [UO₂(SO₄)₂]^2^⁻ complex formation at the sorbent surface, consistent with the observed anion-exchange behavior under acidic conditions^4^. Elemental trends also reveal that F-CMC’s higher uranium capacity corresponds with greater retention of competing ions, whereas F-GG exhibits sharper discrimination against non-target ions, reflecting their distinct functional group distributions^65^. The EDX data corroborate the sorption capacity and selectivity trends observed experimentally, highlighting F-CMC’s higher loading efficiency and F-GG’s enhanced ion selectivity. Detailed spectral interpretation and full elemental compositions are provided in Section B.6 of the Supplementary Information.

#### Testing on acidic leachate

**Fig. 7:**
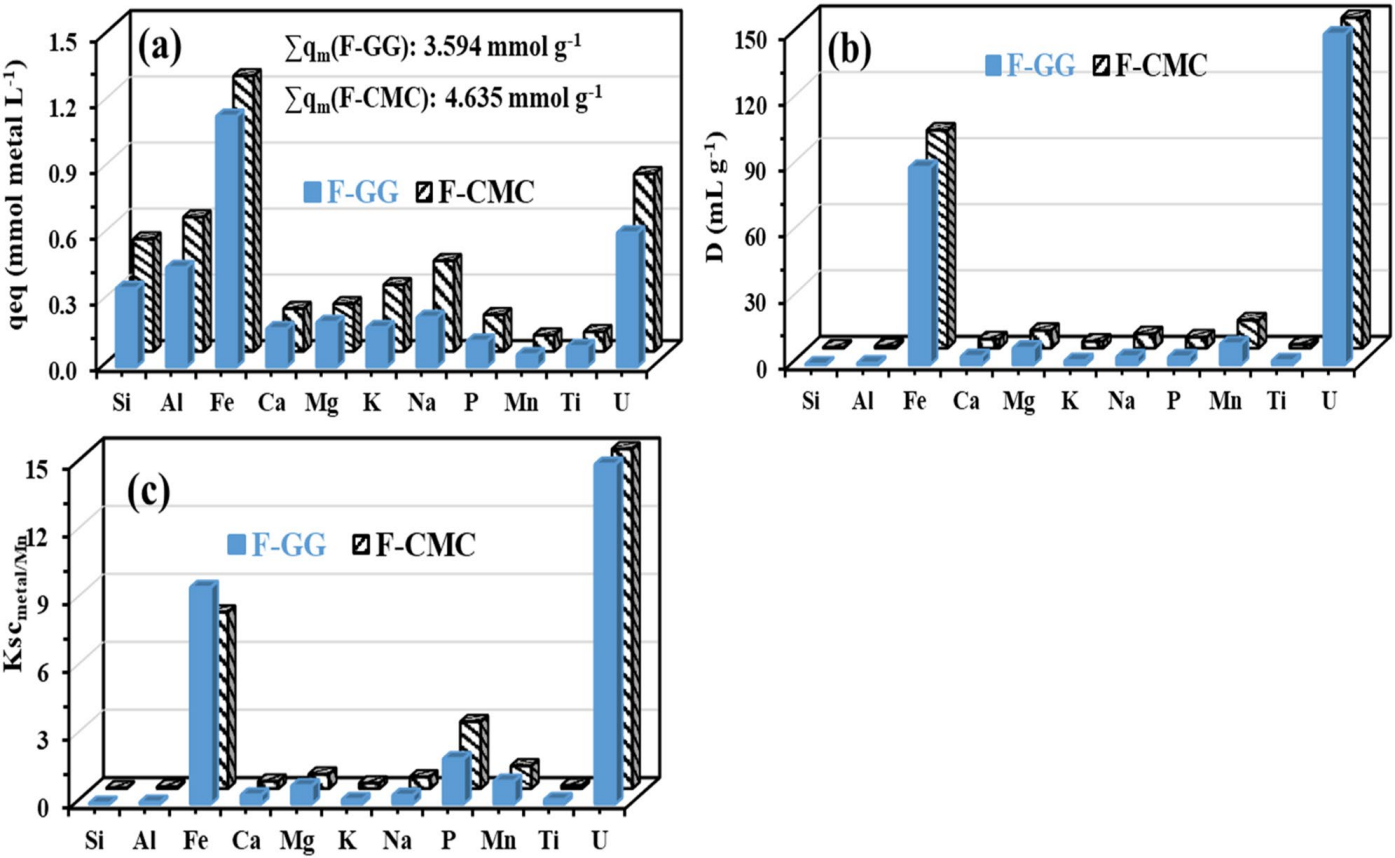
Selectivity tests: (**a**) sorption capacities (in mmol metal/g), (**b**) D (in mL/g), and (**c**) *SC*_metal/Ti_ (× 10^3^). (pH_0_: 4.01, SD: 0.5 g/L, Time: 3 h, Temperature: 27 ± 1 °C, 200 rpm).

Sorption of uranium experiments were conducted under controlled conditions (pH_0_: 4.0, SD: 0.5 g/L, contact time: 120 min, room temperature: 27 ± 1 °C, agitation speed: 200 rpm). Post-equilibrium and phase separation, F-CMC and F-GG sorbents were evaluated for their ability to recover uranium from acidic ore leachates derived from Egyptian ores obtained at the El-Sella mining deposit^34^. Their sorption performance was compared using three key metrics (Fig. 7): equilibrium capacity (q_eq_, a), distribution ratio (D, b), and selectivity coefficient (Ksc_metal/Mn_ = D_metal_/D_Mn_, c)^18^. Even with an overwhelming presence of competing metal ions, both sorbents exhibited exceptional affinity for U(VI), with capacities of 0.609 mmol/g for F-GG (⁓145.21 mg/g) and 0.807 mmol/g for F-CMC (⁓192.17 mg/g). These values are generally higher than those observed for other metal ions but slightly lower than those obtained from synthetic solutions, which are 0.713 mmol/g for F-GG and 1.119 mmol/g for F-CMC (Fig. 7a). Notably, performance disparities between complex leachates and synthetic solutions were consistent: F-GG showed a ≤ 14.65% reduction in capacity, while F-CMC experienced up to 27.86% decline. This underscores F-GG’s superior resilience in multi-ion matrices, highlighting its potential for selective uranium extraction from real-world leachates. The total sorption capacities (in mmol⋅metal/g) followed the order: F-CMC (*∑*qm): 4.635 (⁓340.88 mg/g) > F-GG (*∑*qm): 3.594 (⁓265.76 mg/g), significantly exceeding values for synthetic solutions (F-CMC: 1.119 mmol/g and F-GG: 0.713 mmol/g). This enhancement likely stems from competitive ion effects and multisite interactions with co-metal ions, implying that abundant functional groups critically enable multi-metal uptake^13^.

The distribution ratio analysis reveals that F-CMC exhibits superior UO_2_^2+^ affinity with a D value of 1102.96 mL/g, significantly exceeding F-GG’s 732.50 mL/g by 1.53-fold (Fig. 7b). Both sorbents demonstrate remarkable selectivity, with UO_2_^2+^ distribution ratios 7.8–9.1 times higher than competing metal ions (F-GG: 121.38 mL/g; F-CMC: 141.08 mL/g). Among the non-target species, Fe(III) displays secondary affinity but remains substantially lower than UO_2_^2+^, confirming the preferential binding of uranyl ions^8^. The selectivity coefficients (Ksc_metal/Mn_) further highlight the hierarchy of sorption: U(VI) > > Fe(III) > other metals (Fig. 7c), demonstrating the ability of both materials to discriminate against chemically similar ions. Importantly, this high selectivity persists in complex polymetallic leachates, enabling efficient uranium concentration in the solid phase. The EDX analysis (Fig 7) confirms the selective U(VI) sorption by F-CMC and F-GG sorbents after treating acidic ore leachate, confirming strong U(VI) sorption. While residual elements (Na, Al, Si, P, S, K, Ca) remained, U and Fe were sorbed. F-CMC achieved higher capacity (3.2 mmol/g) with broader affinity, whereas F-GG showed lower capacity (2.1 mmol/g) but enhanced UO_2_^2+^ specificity. This complementary behavior allows for optimized leachate treatment strategies.

The selectivity of UO_2_^2+^ for different sorbents is governed by multiple synergistic factors^4,8^: (a) HSAB principle**:** As a hard Lewis acid (Pearson’s classification), UO₂^2^⁺ preferentially coordinates with O- > N- donors, with binding affinity further influenced by chelate ring size, (b) pH-dependent protonation: Below pH 4, carboxylic group protonation (–COOH) reduces electrostatic interactions, (c) Electronic effects: Electron-withdrawing/releasing character of substituents (e.g., phenolic –OH in F-GG vs. methoxy –OCH₃ in F-CMC) modulates O/N donor strength, (d) Steric constraints: Bulky phenolic groups create greater accessibility limitations than smaller methoxy groups, and (e) A slightly higher specific surface area (F-CMC > F-GG) increases available binding sites. These factors enable synergistic UO₂^2^⁺ sorption via dual coordination through amide groups (carbonyl-O and amine-N), pH-tunable electrostatic interactions at deprotonated -COO⁻/-NH sites, and size-selective matrix diffusion. FTIR shifts (1538–1517 cm⁻^1^) and U–O/N vibrations (650–400 cm⁻^1^), along with competitive ion studies, confirm inner-sphere complexation pathways, while steric effects enhance uranyl specificity.

#### Metal desorption and sorbent recycling

Effective metal desorption is critical for sorbent regeneration and economic viability^62^. Both F-CMC and F-GG sorbents exhibited excellent uranium desorption using 0.25 M NaHCO₃ or 0.2 M HCl, reaching equilibrium within 90 min^34^. Over five consecutive cycles (Table S7), both sorbents showed strong reusability, with initial desorption > 96% and complete Cycle 1 regeneration. However, F-CMC exhibited greater capacity decay (ΔSE = 8.26%) than F-GG (ΔSE = 5.35%), revealing a clear trade-off between maximum initial uptake and long-term cycling stability. The observed 5–8% capacity decrease over five cycles is attributed to partial site deactivation, incomplete desorption of strongly chelated uranium, and subtle matrix reorganization during regeneration, as evidenced by FTIR peak broadening (1650–1550 cm⁻^1^). This divergence stems from their distinct binding mechanisms: F-CMC’s strong carboxylate chelation requires harsher elution that can gradually degrade functional groups, whereas F-GG’s robust phenolic/hydroxyl matrix better resists acidic stress, enabling milder regeneration^49^. The minor capacity loss (5–8%) over cycles is attributed to partial site deactivation and subtle polymer reorganization. The UO₂^2^⁺ desorption from F-CMC/F-GG occurs through proton-mediated ion exchange, where HCl-supplied H⁺ ions displace uranium complexes from binding sites (Sorbent–UO_2_^2+^ + 2H^+^ → Sorbent–2H⁺ + UO_2_^2+^)^58^. Acidic leaching effectively desorbs U(VI), and both sorbents exhibit excellent reusability, maintaining stable capacities over five cycles. FTIR analysis (Fig. 1) confirms preserved functional groups, demonstrating their chemical durability and robustness for practical applications.

## Conclusion

Two superabsorbent hydrogels, F-CMC and F-GG, were successfully synthesized via free-radical polymerization and comprehensively characterized. They exhibited exceptional uranium sorption capacities of 269.26 and 169.78 mg g⁻^1^, for F-CMC and F-GG, respectively, at optimal pH 4.0. The sorption process was spontaneous, exothermic, and fit the Langmuir isotherm and pseudo-first-order kinetic models, confirming monolayer chemisorption. Equilibrium was reached within 90–120 min, with hydrogel swelling enhancing mass transfer, slightly impeded by polynuclear uranyl formation. Sorption proceeds via a dual mechanism: ion-exchange of anionic uranyl-sulfate complexes at low pH and chelation by functional groups (amino, hydroxyl, carboxyl) under mild acidity. Effective desorption (≥ 92%) using 0.25 M NaHCO₃ or 0.2 M HCl enabled excellent reusability over five cycles. In practical ore leachates, F-CMC retained superior capacity (⁓0.807 mmol/g), while F-GG demonstrated superior selectivity against competing ions with minimal capacity loss (≤ 14.65%), confirming their efficacy for uranium recovery from acidic mining solutions. While future work should address mechanical property characterization, selectivity optimization in complex matrices, and scalability, these hydrogels present a robust, reusable platform for uranium extraction in environmental remediation and mineral processing.

## Supplementary Information

Supplementary Information.

## Data Availability

All data generated or analyzed during this study are included in this article.

## Abbreviations

CHN: Elemental analysis
SEM: Scanning electron microscop
FTIR: Fourier-transform infrared spectroscopy
TG/DTA: Thermogravimetric analysis
BET: Surface area analysis
EDX: Energy-dispersive X-ray spectroscopy
