# Supplementary material for: Effect of biopolymer structure on uranium sorption by superabsorbent hydrogels based on CMC/guar gum
Source: Sci Rep. 2026 Apr 20;16:12893. doi: 10.1038/s41598-026-46963-3 (PMC13096171; doi:10.1038/s41598-026-46963-3)
Supplement: Supplementary file 1 — Supplementary Information. [file 41598_2026_46963_MOESM1_ESM.docx]

**Effect of Biopolymer Structure on Uranium Sorption by Superabsorbent Hydrogels based on CMC/Guar Gum**

**Shimaa M.Elsaeed^1,*^, Elsayed G.Zaki^1,2,3^, Ibrahim E.El-Tantawy^3^, Marwa M.Rashad^5^, Ahmed A.Galhoum^5,*^**

^1^ Egyptian Petroleum Research Institute, 11727 Cairo, Egypt.

^2^ Sinai University, Kantra Branch, center for scientific research and sustainable development, Ismailia 41632, Egypt.

^3^ Faculty of Postgraduate Studies for Nanotechnology, Cairo University, El-Sheikh Zayed, Giza 12588, Egypt.

^4^ Chemistry Department, Faculty of Science, Menoufia University, Shebin El-Kom, Egypt.

^5^ Nuclear Materials Authority, P.O. Box 530, El-Maadi, Cairo, Egypt.

*Corresponding Authors: [shy_saeed@yahoo.com](mailto:shy_saeed@yahoo.com) & [galhoum_nma@yahoo.com](mailto:galhoum_nma@yahoo.com)

**Keywords:** **Hydrogels, Carboxymethyl cellulose, Guar gum,** Graft copolymerization, **UO_2_^2+^ sorption,** Sorption i**sotherms and kinetics, Ore leachate application.**

ـــــــــــــــــــــــــــــــــــــــــــــــــــــــــــــــــــــــــــــــــــــــــــــــــــــــــــــــــــــــــــــــــ

***Corresponding authors E-mail:** shymasaeed@epri.sci.eg (Shyma Elsaeed) and [galhoum_nma@yahoo.com](mailto:galhoum_nma@yahoo.com) (Ahmed Galhoum).

**A. Experimentals**

**Table S1.** Sorption modeling: kinetics and sorption isotherms ^1,2^

| Process | Model | Equation | Parameters | |
| --- | --- | --- | --- | --- |
| Kinetics | PFORE | $q(t)=q_{eq,1}(1-e^{k_{1}t})$ | q_eq,1_  (mg U g^-1^) | k_1_  (min^-1^) |
|  | PSORE | $q(t)=\frac{q_{eq,2}^{2}{\times k}_{2} \times t}{1 + q_{eq,2} \times k_{2} \times t}$ | q_eq,2_  (mg U g^-1^) | k_2_  (L mg^-1^ min^-1^) |
|  | sRIDE  (Weber & Morris)  [30] | $q_{(t)} = k_{int., i}. t^{0.5}+C$  Several linear sections corresponding to different regimes of resistance (i) to intraparticle diffusion may co-exist (K_int, i_) (linear regression calculation) | K_int.,i_ (mg g^-1^ min^-0.5^) | |
| Isotherms | Langmuir | $\frac{C_{eq}}{q_{eq}}=\frac{C_{eq}}{q_{max}}+\frac{1}{bq_{max}}$ | q_max_  (mg U g^-1^) | b_L_  (L mg^-1^) |
|  | Freundlich | $q = k_{F} C_{eq}^{1/n}$ | k_F_ | N  (dimensionless) |
|  | Temkin | $q_{eq}= B_{T} {lnC}_{eq} +B_{T} {lnA}_{T}$  Where, $B_{T}= \frac{RT}{b_{T}}$ | A_T_  (L mg^-1^) | b_T_  (J mol^-1^) |

**Table S2**. Elemental composition of ore, acidic leachate (PLS), pH-adjusted PPLS, and post-sorption treated solutions using F-GG/F-CMC sorbents.

| Element | Reference oxide | Wt. (%) | Metal Concentration, mg/L | | C_eq_, (mg/L) | |
| --- | --- | --- | --- | --- | --- | --- |
|  |  | Ore | PLS^a^ | PPLS^b^ | F-GG | F-CMC |
| Si | SiO_2_ | 70.65 | 43090.30 | 42928.30 | 42923.30 | 42921.13 |
| Al | Al_2_O_3_ | 13.24 | 17956.34 | 17897.13 | 17891.04 | 17888.89 |
| Fe | Fe_2_O_3_ | 5.19 | 15784.23 | 744.23 | 712.35 | 709.17 |
| Ca | CaO | 1.81 | 1934.61 | 1908.93 | 1905.46 | 1905.03 |
| Mg | MgO | 0.64 | 664.37 | 660.37 | 657.92 | 657.74 |
| K | K_2_O | 1.28 | 4098.23 | 3899.45 | 3895.97 | 3893.53 |
| Na | Na_2_O | 0.35 | 1138.52 | 1471.52 | 1468.94 | 1466.78 |
| P | P2O5 | 0.68 | 1041.20 | 1039.67 | 1037.87 | 1037.09 |
| Mn | MnO | 0.23 | 328.31 | 321.93 | 320.43 | 319.90 |
| Ti | TiO_2_ | 1.05 | 2537.22 | 2527.87 | 2525.67 | 2525.74 |
| U (mg/L) | 1155.83 | 0.1156 | 299.31 | 270.19 | 197.76 | 174.15 |
| LOI |  | 4.25 |  |  |  |  |
| **Total** |  | **99.48** |  |  |  |  |

^a^: pregnant leaching solution, ^b^: precipitated pregnant leaching solution.

**B. Results and discussion**

**B.1. CHN analysis**

**Table S3.** CHN analysis of the CMC, F-CMC, GG, and F-GG.

| **Sample** | **C (%)** | **H (%)** | **N (%)** | **O* (%)** |
| --- | --- | --- | --- | --- |
| **CMC** | 37.27 | 5.98 | 0.12 | 56.63 |
| **F-CMC** | 36.56 | 5.56 | 10.48 | 47.40 |
| **GG** | 39.89 | 6.95 | 0.88 | 52.28 |
| **F-GG** | 40.21 | 6.79 | 12.46 | 40.54 |

* obtained by difference to 100% (w/w fraction).

**B.2. Zeta-potential measurements**


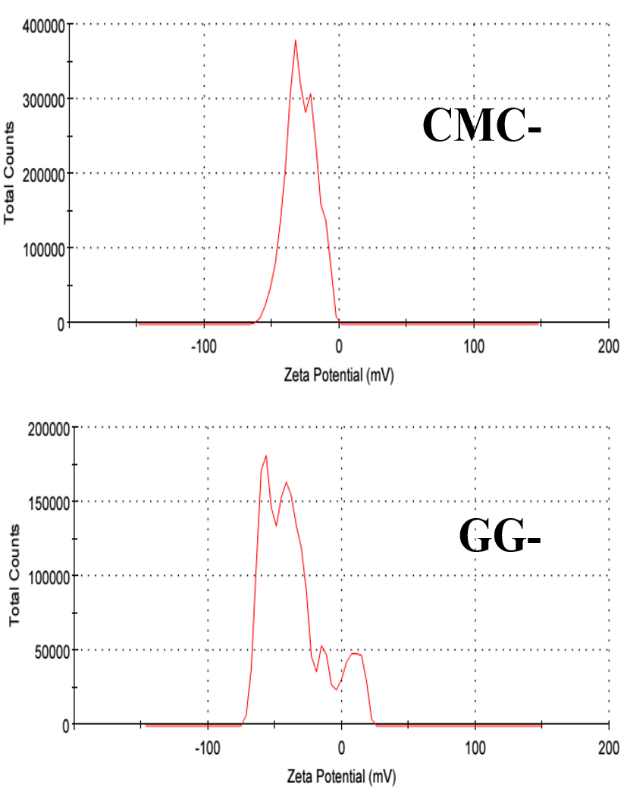

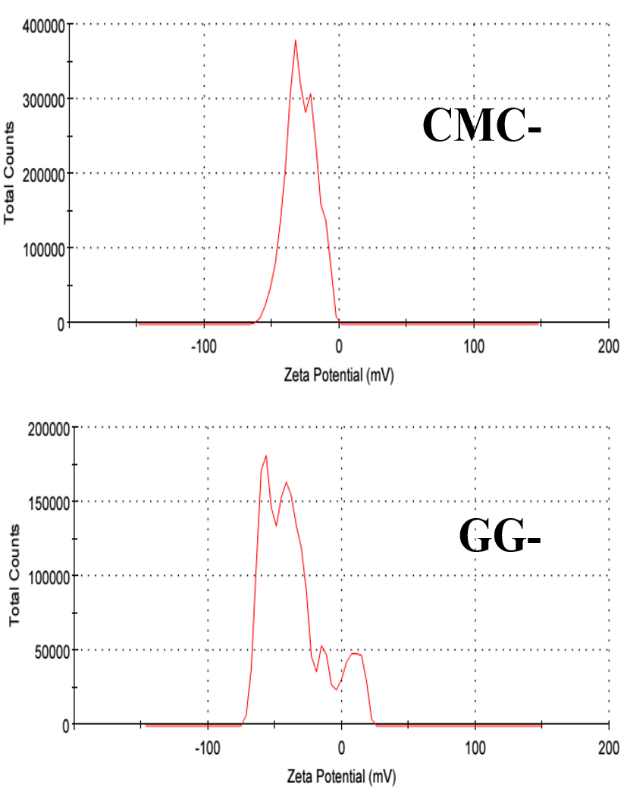


**Fig. S1.** Zeta-potential of F-CMC and F-GG sorbents.

**B.3. Surface area analysis**

**Fig. S2.** Textural characterization of F-CMC and F-GG sorbents.

| **Parameter** | **F-CMC** | **F-GG** |
| --- | --- | --- |
| SSA (m^2^ g^-1^) | 52.162 | 51.115 |
| V_P_ (cm^3^ STP g^-1^) | 0.043590 | 0.015485 |
| Mean pore diameter (nm) | 1.6713 | 6.0589 |

**B.4. Uptake kinetics**

**Fig. S3.** kinetic profiles for PSORE modeling (pH_o_: 4;0; C_o_: 200 mg/L; T: 27 ^o^C; SD: 0.5 mg/L).

**Fig. S4.** kinetic profiles-Multi-linear plots of the sRIDE: q(t) vs. t^(0.5)^.

**Table S4.** Kinetics parameters for UO_2_^2+^ ions sorption.

| **Model** | **Sorbent** | **K_1_×10^-3^ (min^-1^)** | **q_eq_ (mg/g)** | **R^2^** |
| --- | --- | --- | --- | --- |
| **PFORE** | **F-CMC** | 32.24 | 257.99 | 0.9946 |
|  | **F-GG** | 37.54 | 161.88 | 0.9984 |
| **Model** | **Sorbent** | **K_2_×10^-4^ (mg/g.min)** | **q_eq_ (mg/g)** | **R^2^** |
| **PSORE** | **F-CMC** | 2.000 | 294.12 | 0.9868 |
|  | **F-GG** | 4.172 | 181.82 | 0.9922 |
| **Model** | **Sorbent** | **K_id,1_ (mg/g.min^0.5^)** | **K_id,2_ (mg/g.min^0.5^)** | **K_id,3_ (mg/g.min^0.5^)** |
| **sRIDE** | **F-CMC** | 28.862 | 19.315 | 0.3555 |
|  | **F-GG** | 20.789 | 9.067 | 0.3513 |

**B.5. Sorption isotherms and thermodynamics**

**Fig. S5a.** Linearization of Langmuir plots using F-CMC, and F-GG sorbents.

**Fig. S5b.** Linearization of Freundlich plots for UO_2_^2+^ sorption using F-CMC, and F-GG sorbents.

**Fig. S5c.** Linearization of Temkin plots for UO_2_^2+^ sorption using F-CMC, and F-GG sorbents. (pH_o_: 4.0; T: 25 ^o^C; SD: 0.5 g L^-1^; Time: 4 h).

**Table S5.** Isotherm constants for UO_2_^2+^ sorption.

| **Sorbent** | T (K) | Q_max.Exp._  mg/g | Langmuir isotherm | | | Freundlich isotherm | | | Temkin isotherm | | |
| --- | --- | --- | --- | --- | --- | --- | --- | --- | --- | --- | --- |
|  |  |  | Q_max._ | *b*_L_×10^3^  (L/mg) | R^2^ | 1/n | *K_F_,*  (mg/g) | R^2^ | A_T_,  (L/mg) | b_T_,  kJ/mol) | R^2^ |
| **F-CMC** | 300 | 266.26 | 277.78 | 93.51 | 0.997 | 0.382 | 47.9513 | 0.949 | 1.5459 | 50.610 | 0.895 |
|  | 308 | 254.16 | 270.27 | 70.88 | 0.995 | 0.394 | 38.2737 | 0.959 | 1.0110 | 49.010 | 0.863 |
|  | 318 | 243.98 | 263.16 | 55.72 | 0.990 | 0.434 | 32.5762 | 0.917 | 0.7173 | 53.243 | 0.853 |
|  | 328 | 238.70 | 256.41 | 50 | 0.987 | 0.442 | 29.8882 | 0.915 | 0.6230 | 52.902 | 0.848 |
| **F-GG** | 300 | 169.28 | 181.82 | 52.28 | 0.995 | 0.356 | 28.9734 | 0.973 | 0.9513 | 31.964 | 0.932 |
|  | 308 | 163.72 | 178.57 | 45.71 | 0.996 | 0.391 | 23.7192 | 0.973 | 0.6578 | 33.265 | 0.943 |
|  | 318 | 159.38 | 175.44 | 40.23 | 0.994 | 0.412 | 20.6016 | 0.966 | 0.5244 | 33.922 | 0.930 |
|  | 328 | 152.68 | 169.49 | 36.31 | 0.993 | 0.440 | 17.2942 | 0.957 | 0.4178 | 34.352 | 0.927 |

**B.6. Uranyl interaction mechanism**

**B.6.1. FTIR characterization**

FTIR spectroscopy elucidated UO₂²⁺ binding interactions by identifying reactive functional groups (Fig. 1). Spectral changes, including band shifts, intensity variations, appearance, and/or disappearance of bands, indicated binding-induced modifications. Both sorbents exhibited subtle changes in broad overlapping bands corresponding to –OH, >C=O, and –NH stretching vibrations, suggesting minor structural rearrangements upon uranium sorption.

For F-CMC, the spectrum after UO_2_^2+^ sorption displayed reduced broadness and intensity, along with red-shifts in key vibrational bands:

- The –OH/–NH₂ stretching band shifted from **3434 cm^-1^ to 3442 cm^-1^**, while the –CH₂ asymmetric/symmetric stretches (**2939/2864 cm^-1^**) shifted to **2968/2936 cm^-1^**, indicating altered hydrogen bonding environments due to metal coordination ^3^.
- The carbonyl (>C=O) band at **1732 cm^-1^** shifted to **1731 cm^-1^**, while the amide II band (N–H bending/C–N stretching) at **1552 cm^-1^** moved to **1538 cm^-1^**, both with increased sharpness, confirming UO_2_^2+^ interaction with carbonyl and amide functionalities.
- Perturbations in the fingerprint region (**1459–1062 cm^-1^**) suggested ligand-specific interactions ^4^:
- The **1459 cm^-1^** band (assigned to –CH₂/–CH₃ bending and N–H vibrations) indicated UO₂²⁺ coordination with secondary amines.
- Bands at **1280–1062 cm^-1^** (C–O/C–N stretches) reflected modifications in carboxylate/amide groups due to chelation.
- **Uranyl-specific vibrations** were observed at **922, 861, and 836 cm^-1^**, corresponding to UO_2_^2+^ asymmetric/symmetric stretches (ν₃/ν₁), confirming coordination with oxygen-donor ligands (e.g., carboxylate, hydroxyl, or amine groups) ^5^.
- Low-frequency bands at **650 and 530 cm^-1^** suggested U–O/N bond formation, likely via amidoxime-N or carboxylate-O coordination ^6^.
- The disappearance of peaks at **1117, 1051, 787, and 614 cm^-1^** indicated structural reorganization in the polymer matrix due to UO_2_^2+^-induced bond reconfiguration ^7^.

For F-GG, the FTIR spectra analysis revealed distinct spectral changes post-sorption. The k**ey observations are:**

- The broad O–H/N–H stretching band (~3425 cm⁻¹) blue-shifits to 3419 cm⁻¹ with reduced intensity, suggesting UO_2_^2+^ coordination with –OH/–NH groups. Minor shifts in C–H peaks (2932, 2891 cm^-1^) indicate indirect effects from UO_2_^2+^ binding to adjacent polar groups.
- The carbonyl region exhibited weakened interactions:
- Bands at 1676 and 1650 cm⁻¹ **(>C=O)** shifted to 1678 and 1646 cm^-1^, respectively.
- The **amide II/C–N stretch (1537 cm^-1^)** moved to 1539 cm^-1^, with a new band emerging at 1517 cm^-1^, while the band at 1457 cm⁻¹ (–CH₂/–CH₃ bending/N–H vibrations) moved to 1456 cm^-1^, confirming UO_2_^2+^ coordination with nitrogen/oxygen sites.
- **Key fingerprint region observations (1500–400 cm^-1^):**
- Minor blue shifts (e.g., **1210 to 1214 cm^-1^, 1038 to 1040 cm^-1^**) indicated electronic/conformational changes in C–O/C–N bonds.
- New bands at **1185, 677, 670, and 422 cm^-1^** provided critical binding insights:
- **at 1185 cm^-1^:** Perturbed C–O/C–N stretches (e.g., carboxylate/amidoxime) due to UO₂²⁺ coordination.
- **at 677–670 cm^-1^:** U–O/N covalent bonds, confirming inner-sphere complexation.
- at **422 cm^-1^:** U–O bending modes, characteristic of uranyl complexes.
- The **919 cm^-1^** band (UO_2_^2+^ asymmetric stretch) further validated oxygen-donor coordination ^5^.
- The disappearance of **869 cm^-1^** (C–O–C/C–O stretching in glycosidic linkages or carboxylate groups) and 511cm^-1^ (polysaccharide ring deformations ^7^) confirmed UO_2_^2+^-driven structural reorganization.

FTIR analysis confirms effective UO_2_^2+^ binding to F-CMC and F-GG sorbents via: 1) **Coordination and hydrogen bonding**, evidenced by shifted –OH/–NH bands (**3400–3200 cm^-1^**), b) **Direct chelation**, demonstrated by new U–O/N vibrations (**650–400 cm^-1^**), c) **Structural modifications**, reflected in altered C–O/C–H regions (**1800–1000 cm^-1^**), and d) the disappearance of native bands and emergence of uranyl-specific peaks validated binding through **covalent (U–O/N), electrostatic (–COO⁻), and hydrogen-bonding interactions**, supporting both **inner- and outer-sphere complexation mechanisms ^8^**.

**B.6.2. Detailed EDX spectra and elemental composition analysis**

Energy-dispersive X-ray (EDX) analysis of spent F-CMC and F-GG sorbents after acidic ore leachate treatment revealed significant uranium uptake (Fig. S6 & Table S6). Both sorbents showed characteristic peaks for matrix elements (Na, Al, Si, P, S, K, Ca) but no detectable Fe signals, suggesting competitive displacement during U(VI) sorption. The EDX spectra after UO₂²⁺ sorption revealed characteristic uranium signals at 3.17 keV (Mα₁), 13.44 keV (Lα₂), and 13.62 keV (Lα₁)^9^, confirming successful uranium sorption. Quantitative analysis measured uranium content. F-CMC shows remarkable uranium sorption (21.46 wt%), outperforming F-GG (6.05 wt%) by 3.5× times, confirming F-CMC's superior sorption capacity, consistent with their respective sorption capacities. Concurrently, the detection of iron (Fe Kα at 6.403 keV: Iron (Fe) appears post-treatment (7.54 wt% in F-CMC, 5.97 wt% in F-GG), suggesting ore-derived Fe sorption) and sulfur signatures (S Kα₂ at 2.31 keV and S Lα at 0.149 keV) from the sulfate medium indicates the presence of uranyl-sulfate complexes. These findings suggest a dual sorption mechanism: a) direct UO₂²⁺ coordination to protonated amine groups and b) anion exchange of SO₄²⁻ species ^10,11^. The coexistence of uranium and sulfate signals implies potential [UO₂(SO₄)₂]²⁻ complex formation at the sorbent surface, consistent with the observed anion-exchange behavior under acidic conditions.

Furthermore, both F-CMC and F-GG showed significant **K** (Kα signal at ~3.31 keV) before treatment (F-CMC: 41.77 wt%; F-GG: 72.13 wt%), and the complete post-sorption disappearance confirms the role of K in **ion exchange** with UO₂²⁺/Fe³⁺/Al³⁺ from the leachate. F-CMC exhibits dramatic Al enrichment (32.84 wt% *vs* F-GG's 10.81 wt%), indicating possible Al-U co-sorption. F-GG showed a significant Ca, Kα at 3.69 keV, increase (4.99 to 27.47 wt%), while F-CMC had minimal change (4.90 to 5.55 wt%), suggesting different binding mechanisms ^12^. Concurrent decreases in Ca²⁺ (∼40%) and K⁺ (∼35%) concentrations indicate ion exchange participation in the sorption mechanism ^12^. Notably, F-CMC demonstrated 3.3× higher uranium uptake than F-GG but with reduced selectivity, as evidenced by greater retention of competing elements (Al³⁺, Ca²⁺). Conversely, F-GG showed sharper discrimination against non-target ions despite its lower capacity. This capacity-selectivity tradeoff reflects their distinct functional group distributions: F-CMC's higher carboxyl density promotes greater uranium loading, while F-GG's stereospecific binding sites enhance ion selectivity.

**Table S6.** Semi-quantitative EDX analysis of the surface ofF-CMC&F-GG pre-/poet-treatment ore acid leachate of Egyptian ore (weight %).

| **Sorbent** | **F-CMC** | | **F-GG** | |
| --- | --- | --- | --- | --- |
| **Element** | **Before** | **After** | **Before** | **After** |
| **Na** | 3.2 | 8.04 | 3.42 | 7.98 |
| **Al** | 1.82 | 32.84 | 4.99 | 10.81 |
| **Si** | 3.6 | 4.53 | 3.48 | 3.54 |
| **P** | 1.53 | 2.64 | 1.83 | 2.94 |
| **S** | 43.18 | 17.4 | 9.15 | 35.24 |
| **K** | 41.77 | 0 | 72.13 | 0 |
| **Ca** | 4.9 | 5.55 | 4.99 | 27.47 |
| **Fe** | 0 | 7.54 | 0 | 5.97 |
| **U** | 0 | 21.46 | 0 | 6.05 |


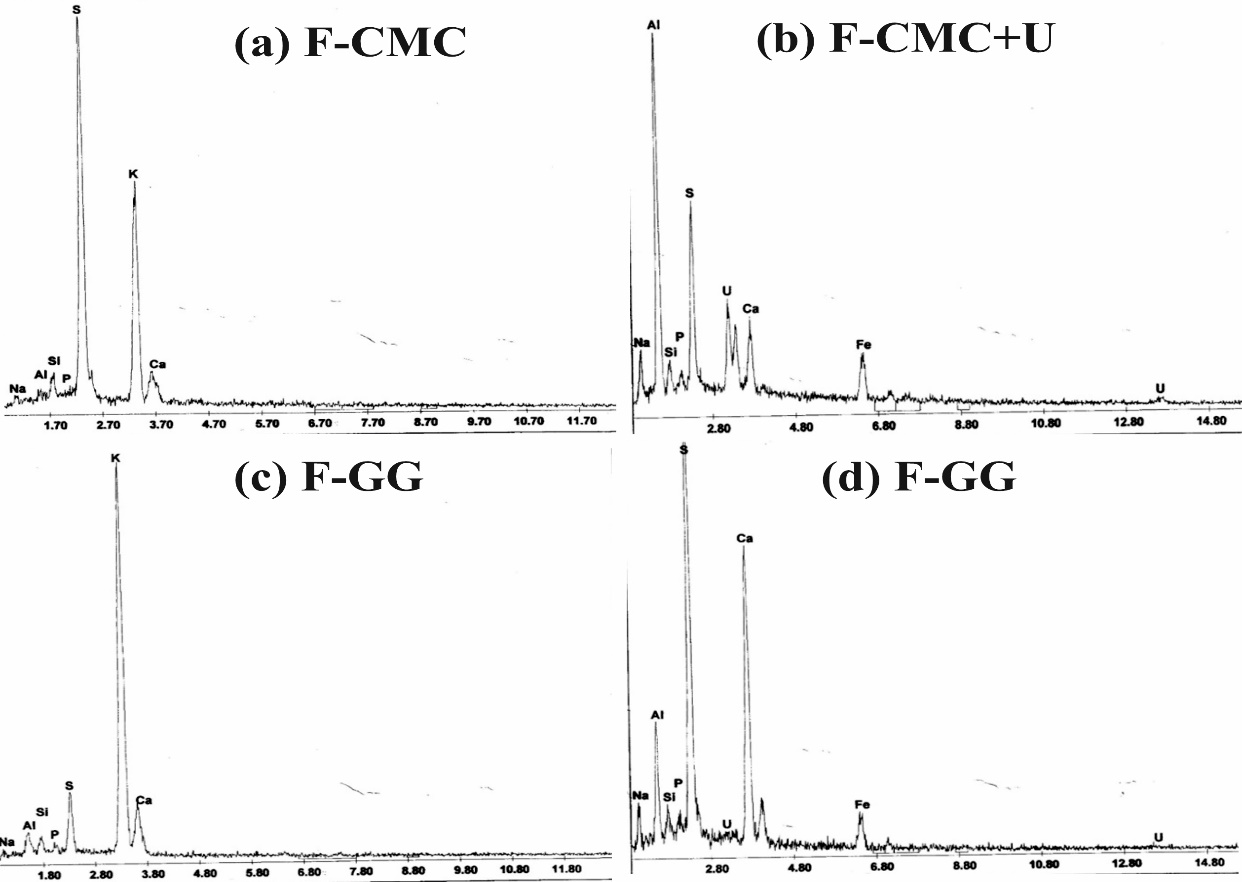


**Fig. S6.** EDX spectra of F-CMC and F-GG sorbents before and after uranium sorption from ore leachate. (pH_0_: 4.06, SD: 0.5 g/L, Time: 3 h, Temperature: 27 ^o^C, 200 rpm).

**B.7. Metal desorption and sorbent recycling**

**Table S7.** Metal desorption and sorbent recycling – relative yields.

| **Sorbent** | **Cycle no.** | **Sorption step** | | | **Desorption step** | | |
| --- | --- | --- | --- | --- | --- | --- | --- |
|  |  | **SE, (%)** | | **Qeq**** | **DE, (%)** | | **Eluate conc.***** |
|  |  | **Average** | **St. Dev.** | **Average** | **Average** | **St. Dev.** | **Average** |
| F-CMC | 1 | 100.0* | 0.00 | 166.84 | 97.10 | 0.73 | 324.00 |
|  | 2 | 96.81 | 0.61 | 161.52 | 95.93 | 0.33 | 320.09 |
|  | 3 | 95.44 | 0.70 | 159.24 | 94.63 | 0.36 | 315.76 |
|  | 4 | 93.56 | 0.57 | 156.10 | 94.40 | 0.92 | 315.00 |
|  | 5 | 92.30 | 0.80 | 154.00 | 92.36 | 0.38 | 308.18 |
| F-GG | 1 | 100.0* | 0.00 | 119.08 | 97.40 | 0.50 | 231.97 |
|  | 2 | 98.09 | 0.13 | 116.80 | 96.46 | 0.52 | 229.75 |
|  | 3 | 97.10 | 0.28 | 115.62 | 95.77 | 0.60 | 228.11 |
|  | 4 | 96.29 | 0.43 | 114.66 | 94.43 | 0.77 | 224.92 |
|  | 5 | 94.28 | 0.53 | 112.26 | 93.32 | 0.34 | 222.27 |

* Reference value of sorption efficiency (at first cycle). (Sorption experiments (C_0_: 108 mg/L, pH_0_: 4.06, SD: 0.5 g/L, Time: 3 h, Temperature: 27 ^o^C, 200 rpm) and desorption experiments ([NaHCO_3_]: 0.25 M; C_0_: 108 mg/L, SD: 1.0 g/L, Time: 1.5 h, Temperature: 27 ^o^C, 200 rpm).

Units: **: mg/g and *** mg/L.

**Reference**

1 Raji, Z., Karim, A., Karam, A. & Khalloufi, S. Adsorption of heavy metals: Mechanisms, kinetics, and applications of various adsorbents in wastewater remediation—A review. *Waste* **1**, 775-805, doi:<https://doi.org/10.3390/waste1030046> (2023).

2 Wang, J. & Guo, X. Adsorption kinetics and isotherm models of heavy metals by various adsorbents: An overview. *Crit. Rev. Environ. Sci. Technol.* **53**, 1837-1865, doi:<https://doi.org/10.1080/10643389.2023.2221157> (2023).

3 Galhoum, A. A. *et al.* Functionalization of poly(glycidylmethacrylate) with iminodiacetate and imino phosphonate groups for enhanced sorption of neodymium - sorption performance and molecular modeling. *React. Funct. Polym.* **180**, 105389, doi:<https://doi.org/10.1016/j.reactfunctpolym.2022.105389> (2022).

4 Mohamed, A., Galhoum, A. A., Saad, A. & Wageh, S. Chitosan/carbon/ZnMn₂O₄ nanocomposite for efficient removal of chromium(VI) and anionic/cationic dyes. *Int. J. Biol. Macromol.*, 146329, doi:<https://doi.org/10.1016/j.ijbiomac.2025.146329> (2025).

5 Sureshkumar, M. K., Das, D., Mallia, M. B. & Gupta, P. C. Adsorption of uranium from aqueous solution using chitosan-tripolyphosphate (CTPP) beads. *J. Hazard. Mater.* **184**, 65-72, doi:<https://doi.org/10.1016/j.jhazmat.2010.07.119> (2010).

6 Imam, E. A. *et al.* Aminophosphonate CuO nanocomposites for uranium(VI) removal: Sorption performance and mechanistic study. *Sep. Purif. Technol.* **323**, 124466, doi:<https://doi.org/10.1016/j.seppur.2023.124466> (2023).

7 Coates, J. *Interpretation of infrared spectra: Apractical Approach. In Encyclopedia of analytical chemistry*. (John Wiley & Sons Ltd, Chochester, 2000).

8 Kam, E., Taşdelen, B. & Osmanlioglu, A. E. Uranyl ion uptake capacity of poly (N-isopropylacrylamide/maleic acid) copolymeric hydrogels prepared by gamma rays. *Radiat. Phys. Chem.* **81**, 618-621, doi:<https://doi.org/10.1016/j.radphyschem.2012.02.041> (2012).

9 El-Seidy, A. M. A. *et al.* Mechanism of uranium(VI) sorption on α-aminophosphonate sorbents: Multimodal spectroscopy and computational study. *RSC Adv.* **15**, 28269, doi:<https://doi.org/10.1039/d5ra04479k> (2025).

10 Al-Ghamdi, A. A. *et al.* Mesoporous magnetic cysteine functionalized chitosan nanocomposite for selective uranyl ions sorption: experimental, structural characterization, and mechanistic studies. *Polymers* **14**, 2568, doi:<https://doi.org/10.3390/polym14132568> (2022).

11 Alghamdi, N. A. Mesoporous magnetic-polyaminated-chitosan nanocomposite for selective uranium removal: performance and mechanistic studies. *Int. J. Environ. Sci. Technol.* **20**, 8319-8340, doi:<https://doi.org/10.1007/s13762-022-04565-2> (2023).

12 Wang, X. L. *et al.* Efficiency and mechanism of adsorption of low concentration uranium in water by extracellular polymeric substances. *J. Environ. Radioact.* **197**, 81-89, doi:<https://doi.org/10.1016/j.jenvrad.2018.12.002> (2019).
